# Responsive Multivesicular Polymeric Nanovaccines that Codeliver STING Agonists and Neoantigens for Combination Tumor Immunotherapy

**DOI:** 10.1002/advs.202201895

**Published:** 2022-06-16

**Authors:** Ting Su, Furong Cheng, Jialong Qi, Yu Zhang, Shurong Zhou, Lei Mei, Shiwei Fu, Fuwu Zhang, Shuibin Lin, Guizhi Zhu

**Affiliations:** ^1^ Center for Translational Medicine Precision Medicine Institute The First Affiliated Hospital Sun Yat‐sen University Guangzhou 510080 China; ^2^ Department of Pharmaceutics and Center for Pharmaceutical Engineering and Sciences; Institute for Structural Biology and Drug Discovery School of Pharmacy; The Developmental Therapeutics Program Massey Cancer Center Virginia Commonwealth University Richmond VA 23298 USA; ^3^ Department of Chemistry University of Miami Coral Gables FL 33146 USA; ^4^ The Dr. John T. Macdonald Foundation Biomedical Nanotechnology Institute University of Miami Miami FL 33136 USA

**Keywords:** cancer immunotherapy, cGAS‐STING, nanovaccine, neoantigen, pH responsiveness, polymeric nanocarrier, vaccine codelivery

## Abstract

Immune checkpoint blockade (ICB) has significantly advanced cancer immunotherapy, yet its patient response rates are generally low. Vaccines, including immunostimulant‐adjuvanted peptide antigens, can improve ICB. The emerging neoantigens generated by cancer somatic mutations elicit cancer‐specific immunity for personalized immunotherapy; the novel cyclic dinucleotide (CDN) adjuvants activate stimulator of interferon genes (STING) for antitumor type I interferon (IFN‐I) responses. However, CDN/neoantigen vaccine development has been limited by the poor antigen/adjuvant codelivery. Here, pH‐responsive CDN/neoantigen codelivering nanovaccines (NVs) for ICB combination tumor immunotherapy are reported. pH‐responsive polymers are synthesized to be self‐assembled into multivesicular nanoparticles (NPs) at physiological pH and disassembled at acidic conditions. NPs with high CDN/antigen coloading are selected as NVs for CDN/antigen codelivery to antigen presenting cells (APCs) in immunomodulatory lymph nodes (LNs). In the acidic endosome of APCs, pH‐responsive NVs facilitate the vaccine release and escape into cytosol, where CDNs activate STING for IFN‐I responses and antigens are presented by major histocompatibility complex (MHC) for T‐cell priming. In mice, NVs elicit potent antigen‐specific CD8^+^ T‐cell responses with immune memory, and reduce multifaceted tumor immunosuppression. In syngeneic murine tumors, NVs show robust ICB combination therapeutic efficacy. Overall, these CDN/neoantigen‐codelivering NVs hold the potential for ICB combination tumor immunotherapy.

## Graphical abstract

Cancer neoantigens and cyclic dinucleotide (CDN) vaccines can improve immune checkpoint blockade (ICB) immunotherapy, but have poor codelivery. Here, pH‐responsive nanovaccines are reported that efficiently codeliver CDNs/antigens to the cytosol of lymph nodal antigen presenting cells, resulting in efficient STING activation, durable antigen presentation, potent CD8^+^ T‐cell responses with memory, reduced tumor immunosuppression, and robust ICB combination tumor therapy.

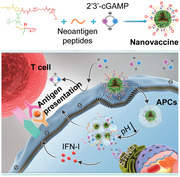

## 1 Introduction

Cancer immunotherapy leverages the host immune system for cancer treatment and has emerged as one of the major therapy modalities. Current cancer immunotherapy approaches range from immune checkpoint blockade (ICB), adoptive T‐cell transfer, oncolytic viruses, to therapeutic vaccines.^[^ ^1^ ^]^ In particular, ICB has shown great potential as a novel and efficacious cancer therapeutic approach, with an increasing number of FDA‐approved ICB agents in the past decade. However, while durable response to cancer immunotherapy relies on sustained antitumor immune responses,^[^ ^2^ ^]^ only a small subset of patients with certain types of cancer durably respond to ICB monotherapies. This is largely due to ICB resistance associated with tumor heterogeneity, sparse antitumor immune cells as ICB targets,^[^ ^3^ ^]^ often low immune checkpoint levels,^[^ ^4^ ^]^ and multitier immunosuppression systemically and locally in the tumor microenvironment (TME).^[^ ^5^ ^]^ Dual ICBs have increased the response rates but also aggravated immune‐related adverse effects due to the loss of immune homeostasis.^[^ ^6^ ^]^ Such challenges demand innovative approaches, such as combination immunotherapy, to maximize clinical benefit of ICB.

Cancer therapeutic vaccines hold the potential to overcome ICB resistance and improve ICB efficacy in combination immunotherapy.^[^ ^6^, ^7^ ^]^ Cancer vaccines can induce innate and/or adaptive antitumor immunity and ameliorate tumor immunosuppression systemically and locally in the TME.^[^ ^8^ ^]^ Conventional cancer therapeutic vaccines have only shown marginal tumor therapeutic efficacy in the clinic so far, largely due to poor codelivery of molecular antigens and immunostimulant adjuvants that is desired for optimal antitumor immunomodulation with minimal immune tolerance. Indeed, so far there are only two cancer therapeutic vaccines used in the clinic: sipuleucel‐T (Provenge) for metastatic prostate cancer (approved by US FDA in 2010) and weakened bacteria Bacillus Calmette–Guérin (BCG) for early stage bladder cancer. Chemically defined tumor‐associated antigen (TAA)^[^ ^9^ ^]^ and neoantigen^[^ ^7^, ^10^ ^]^ peptide vaccines can elicit cancer‐specific immunity, and are attractive for easy manufacturing, cold‐chain‐free transportation and storage, and long shelf‐life. Due to the host central and peripheral immune tolerance, TAAs have by large shown limited tumor therapeutic efficacy in the clinic thus far. By contrast, neoantigens hold the potential to address this limitation to induce potent cancer immunotherapy with minimal immune tolerance because neoantigens are generated from somatic genetic events (e.g., exogenous viral gene integration, somatic mutation, and gene rearrangement) only in cancer cells but not in healthy cells.^[^ ^10a^ ^]^ Thus, neoantigens vaccines can bypass the host central or peripheral immune tolerance to elicit or augment neoantigen‐specific antitumor immunity with strong neoantigen binding.^[^ ^7^ ^]^ Further, although the endogenous neoantigens elicit neoantigen‐specific T cells, such endogenous neoantigen‐specific T cells are extremely rare (0.002–0.4% in melanoma patients).^[^ ^11^ ^]^ This makes neoantigen vaccines desirable to expand these cell repertoires to improve tumor therapeutic efficacy.^[^ ^10a^ ^]^

The overall poor immunogenicity of peptide antigens demands the use of potent immunostimulants as adjuvants of peptide vaccines, in which the adjuvants elicit innate immunity and potentiate the immunogenicity of peptide antigens, whereby eliciting potent and long‐lasting (with memory) cancer‐specific adaptive immunity.^[^ ^12^ ^]^ Among various types of immunomodulators, agonists for the cyclic GMP‐GMP (cGAMP) synthase (cGAS)‐STING (cGAS‐STING) signaling pathway is an emerging class of potent immunostimulant adjuvants that hold great potential for cancer immunotherapy.^[^ ^13^ ^]^ cGAS‐STING pathway plays a critical role in controlling the transcription of a series of host immune defense genes. Upon activation by CDNs (e.g., cGAMP), STING triggers IFN‐I responses that further activate multifaceted downstream proinflammatory responses.^[^ ^14^ ^]^ This allows the maturation of APCs to promote tumor antigen presentation and the priming of antitumor T cells.^[^ ^15^ ^]^ Moreover, cGAS‐STING activation in the TME can also turn an immunosuppressive “cold” TME to a proinflammatory “hot” TME, the latter of which is critical for effective immunotherapy of solid tumors. These features make STING agonists appealing immunostimulants for cancer immunotherapy.^[^ ^15^, ^16^ ^]^

The optimal antitumor adaptive immunomodulation often requires adjuvant/antigen codelivery to lymphoid tissues [e.g., lymph nodes (LNs) and spleens] and APCs,^[^ ^17^ ^]^ where molecular vaccines are released and versatile antitumor immunity is orchestrated.^[^ ^18^ ^]^ However, efficient codelivery of neoantigen peptides and STING agonists such as CDNs have been limited by 1) the negative charges, susceptibility to enzymatic degradation, and hydrophilicity of CDNs, 2) the neoantigen peptide heterogeneity of electrostatic charges and water solubility, and 3) the resulting typically short half‐lives, poor pharmacokinetics, and limited bioavailability of CDNs and neoantigen peptides. NVs hold great potential to efficiently codeliver peptide antigens and immunostimulant adjuvants^[^ ^19^ ^]^ to lymphoid tissues and APCs. Moreover, NVs can also prevent the random dissemination of vaccines, especially adjuvants, which otherwise cause severe and sometimes fatal immune toxicity including systemic cytokine storm syndromes due to acute elevation of systemic inflammatory cytokines. In addition to stable vaccine loading in NVs with minimal premature vaccine leaking prior to uptake into APCs, vaccine molecules need to be effectively released once uptake by APCs, in which adjuvants can activate intracellular immune receptors (e.g., STING for CDNs) and antigens can be proteolytically processed (except for minimal peptide epitopes) and complexed with major histocompatibility complexes (MHC) for antigen presentation. Therefore, approaches to enhancing the intracellular vaccine release from stable NVs in APCs are highly desired for the optimal immunomodulatory efficacies with minimal immunotoxicity.^[^ ^20^ ^]^ A number of CDN delivery systems have been reported using various nanoparticles such as liposomes,^[^ ^14^, ^21^ ^]^ polymeric particles,^[^ ^22^ ^]^ hydrogel,^[^ ^23^ ^]^ and inorganic materials.^[^ ^22b^ ^]^ For example, liposomal cGAMP reprogrammed macrophages from immunosuppressive M2‐like phenotype toward immunoactivating M1‐like phenotype.^[^ ^21a^ ^]^ However, few of current NVs have efficiently codelivered CDNs and neoantigens that are conditionally released inside APCs to elicit robust and durable tumor‐specific T‐cell immunity.

**Scheme 1:**
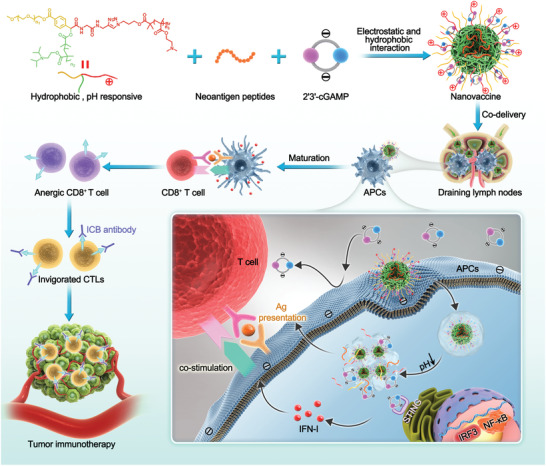
pH‐responsive multivesicular polymeric nanovaccines (NVs) for the codelivery of STING agonists and neoantigens in combination tumor immunotherapy. The star‐shaped polymers were self‐assembled into pH‐responsive nanoparticles (NPs) that coloaded cGAMP and neoantigen peptides through electrostatic and hydrophobic interactions, respectively. The NVs efficiently codelivered cGAMP and neoantigens to the draining lymph nodes (LNs) and intranodal antigen presenting cells (APCs). Upon cell internalization by endocytosis, NVs were disassembled in response to the acidity in the endosome and escaped to the cytosol for STING activation by cGAMP and neoantigen presentation by major histocompatibility complex (MHC). NVs elicited sustained antigen presentation, potentiated and prolonged the neoantigen‐specific T‐cell responses, and ameliorated the tumor immunosuppression in the tumor microenvironment (TME). As a result, the combination of these NVs with immune checkpoint blockade (ICB) showed robust tumor therapeutic efficacy in multiple tumor models.

To address these challenges, we designed polymeric NVs for efficient codelivery and pH‐responsive intracellular release of cGAMP and tumor neoantigens for combination tumor immunotherapy (**Scheme** **1**). We synthesized a series of star‐shaped polymers^[^ ^24^ ^]^ with three arms: 1) cationic poly((2‐dimethylaminoethyl) methacrylate) (PDMA) with a series of lengths that allows electrostatic complexation with negatively charged cGAMP in the self‐assembled NVs, 2) polyethylene glycol (PEG) that shields NP surface charge and enhances the biocompatibility of NPs, and 3) a pH‐responsive poly(2‐(diisopropylamino)ethyl methacrylate) (PDPA) as the hydrophobic core of NPs for the loading of hydrophobic neoantigens via hydrophobic interactions. Upon uptake into the acidic endosome, this pH‐responsive PDPA became protonated, leading to NV disassembly and hence the release of cGAMP and neoantigens. Meanwhile, PDPA protonation is also expected to promote the endosomal escape of 1) cGAMP that needs to bind with and activate cytosolic STING, and 2) MHC‐I‐restricted neoantigens that need to be processed by proteases and complexed with MHC‐I in the cytosol prior to antigen cross‐presentation to elicit CD8^+^ T‐cell responses.^[^ ^25^ ^]^ Further, cGAMP delivered by NVs triggered IFN‐I‐driven inflammation to drive neoantigen cross‐presentation and reprogram the tumor immune microenvironment. The NVs efficiently codelivered cGAMP and neoantigens to draining LNs and the intranodal APCs, elicited potent and long‐lasting (with memory) systemic neoantigen‐specific T‐cell responses, and reprogrammed the immune milieu in LNs and tumor microenvironment that promote antitumor immunomodulation. As a result, these NVs showed potent immunotherapeutic efficacy in multiple mouse tumor models in combination with ICB.

## 2 Results and Discussion

### 2.1 Synthesis of pH‐Responsive Polymeric Micelles as Nanocarriers for cGAMP

**Figure 1:**
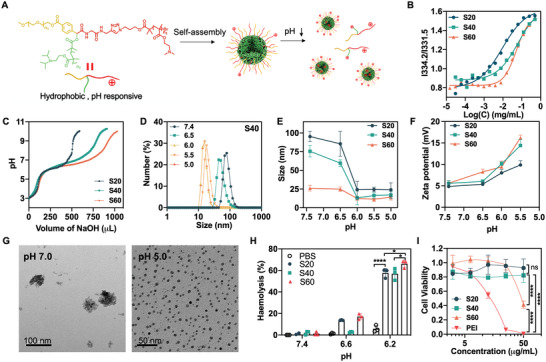
Characterization and screening of pH‐responsive star‐shaped polymer nanoparticles (NPs). A) Schematic illustration of the self‐assembly of star‐shaped multifunctional polymers into pH‐responsive NPs. B) Titration plots for the critical micelle concentration (CMC) measurement for S20, S40, and S60 NPs via the ratios of pyrene fluorescence intensities at 334.2 nm over 331.5 nm (I334.2/I331.5). C) pH titration of NPs (1 mg mL^−1^) using NaOH (0.01 m). D) DLS results showing the hydrodynamic sizes of S40 NPs (1 mg mL^−1^) at different pH conditions. E,F) The hydrodynamic sizes (E) and zeta potential (F) of NPs at the range of pH 5.5 – pH 7.4 indicate the NPs disassembly and the increased electrostatic charge on NPs in response to acidity. G) TEM images of S40 NPs at pH 7.0 and pH 5. The large NP sizes at pH 7.4 and the smaller NP sizes at pH 5 showed NP disassembly at pH5. H) pH‐dependent erythrocyte membrane destabilization by NPs, as measured using an erythrocyte hemolysis assay. Percent hemolysis was calculated relative to H_2_O. I) MTT assay results showed the cell viability of DC2.4 cells treated with different NPs for 24 h. Polyethylenimine (PEI) was used as a positive control. In H and I, data represent mean ± SD (*n = 3*); ns: nonsignificant, *p < 0.05, and ****p < 0.0001 (Student's *t*‐test).

We designed and synthesized star‐shaped pH‐responsive polymers (structures in Scheme 1) that were self‐assembled into multivesicular micellular NPs (**Figure** **1**A) for cGAMP/neoantigen codelivery. First, we synthesized a dual end‐functionalized PEG(‐alkynyl)‐Br via Passerini three‐component reaction.^[^ ^24^ ^]^ Then, at the end of PEG(‐alkynyl)‐Br (Figures S1 and S2, Supporting Information), the pH‐responsive poly(2‐(diisopropylamino)ethyl methacrylate) (PDPA) was synthesized through atom transfer radical polymerization (ATRP) by using PEG(‐alkynyl)‐Br as an initiator, and the hydrophilic and cationic poly(2‐(dimethylamino)ethyl methacrylate (PDMA) was conjugated through click chemistry. The products were measured by ^1^H NMR, Fourier transform infrared (FTIR) (Figure S2, Supporting Information), and GPC (Table S1, Supporting Information). To optimize the cGAMP loading capacity in polymeric NPs, we designed and synthesized the star‐shaped polymers with a series of different lengths of cationic PDMA with the average polymerization degrees of 20, 40, and 60. We named the resulting PEG(‐*g*‐PDMA)‐*b*‐PDPA polymers as S20, S40, and S60. Using pyrene as a hydrophobic fluorescence probe, we determined the critical micelle concentration (CMC) of S20, S40, and S60 to be 0.66, 5.21, and 11.01 µg mL^−1^, respectively (Figure 1B). As shown by acid–base titration, all the three polymers showed good buffering capacities in the pH range of 5 to 7 (Figure 1C), and the elongation of the PDMA chain enhanced the buffering capacity. This can be explained by that the elongation of PDMA enhanced the abundance of protonation at acidic conditions. The p*K*_a_ values, as measured as the pH value in the middle of the two equivalence points in titration curves, were 6.19, 6.68, and 6.55 for S20, S40, and S60, respectively. Next, we demonstrated the sensitive pH responsiveness of these polymer NPs, as shown by their acidic pH‐responsive decrease of hydrodynamics sizes and increase of zeta potentials (Figure 1D: S40 NPs at different pH values; Figure 1E,F and Figure S3 (Supporting Information): all three NPs). Note that, presumably due to the increased water solubility of long PDMA chains, at physiological pH7.4, the NP sizes reduced from ≈95 nm in diameter for S20 to ≈75 nm for S40 and ≈26 nm for S60. The morphology of S40 micelles at pH 7.0 and acid pH 5 were examined by TEM, which verified the NP disassembly in response to acidic pH (Figure 1G). Intriguingly, TEM also revealed the multivesicular micellular substructures of these NPs, likely resulting from the amphiphilic structure of these star‐shaped polymers. Lastly, we studied the ability of these polymers for pH‐responsive endosome escape using an erythrocyte hemolysis assay that is a commonly used to predict the endosomolytic activity of drug carriers.^[^ ^26^ ^]^ The NPs showed a pH‐dependent erythrocyte membrane destabilization (Figure 1H and Figure S4, Supporting Information). Specifically, at pH 6.2, all three NPs showed significant hemolysis, in contrast to negligible hemolysis of these NPs at physiological pH7.4, and moderate hemolysis for only S20 and S60. Moreover, the elongation of the hydrophilic PDMA segment increased the hemolytic activity at pH6.2. These results clearly demonstrated the sensitive pH responsiveness of these NPs at the range of acidic pH that simulate the pH range in the endosome. Such sensitive pH responsiveness is expected to ensure stable vaccine loading in the NPs at physiological pH while facilitating the vaccine release from NPs in the endosome as well as the endosome escape of vaccines to the cytosol. In an MTT cell viability assay, all of these NPs showed low cytotoxicity in DC2.4 dendritic cells (DCs) at up to 50 µg mL^−1^, in contrast to the high cytotoxicity of polyethylenimine (PEI), a commonly used gene delivery carriers as positive control (Figure 1I).

### 2.2 DC Immunostimulation by cGAMP‐Loaded NPs (NP/cGAMP)

**Figure 2:**
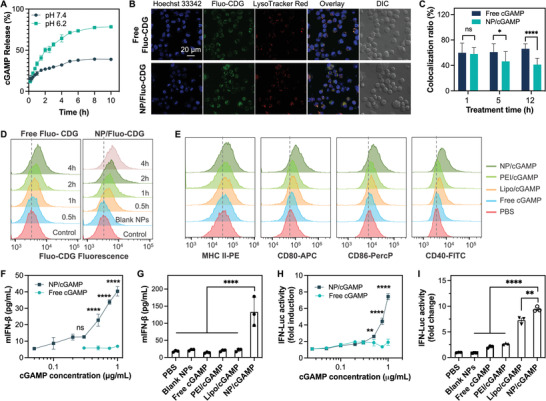
Characterization of nanoparticle (NP)/cGAMP. A) pH‐responsive cGAMP release kinetics from S40 polymeric NPs. B) Confocal microscopy images showed the uptake of free Fluo‐CDG or NP/Fluo‐CDG (1 µg mL^−1^) into DC2.4 cells after a 5‐h incubation. DIC: differential interference contrast. C) Colocalization ratios of Fluo‐CDG with endolysosome as quantified from 20 random cells in confocal microscopy results. D) Flow cytometry results showing the time‐dependent intracellular delivery of free Fluo‐CDG or/Fluo‐CDG in DC2.4 cells (Fluo‐CDG: 1 µg mL^−1^). E) Flow cytometry results showing that, relative to controls, NP/cGAMP enhanced the upregulation of MHC‐II and costimulatory factors CD40, CD80, and CD86 in DC2.4 cells. F) ELISA results showing that NPs significantly promoted the ability of cGAMP to induce murine IFN‐*β* (mIFN‐*β*) in DC2.4 cells after a 24‐h treatment in a dose‐dependent manner. G) NP/cGAMP outperformed controls to induce mIFN‐*β*) in DC2.4 cells (cGAMP: 1 µg mL^−1^, 24‐h treatment). H,I) Fold changes of interferon (IFN) dependent reporter signals in RAW‐ISG cells treated with cGAMP formulations demonstrated the dose‐dependent cGAMP‐selective IFN response (H) (24‐h treatment) and the superior INF induction ability of NP/cGAMP than controls (I) (cGAMP: 1 µg/mL, 24‐h treatment). Lipo/cGAMP: Lipofectamine2000‐transfected cGAMP. PEI/cGAMP: PEI‐transfected cGAMP. NP/cGAMP: cGAMP‐loaded S40 NPs. Data: mean ± SD (*n = 3*); ns: nonsignificant, *p < 0.05, **p < 0.01, and ****p < 0.0001 (Student's *t*‐test).

To develop these multivesicular polymeric NPs as vaccine carriers, we then studied their cGAMP loading via electrostatic interactions between cationic PDMA in NPs and anionic cGAMP. We measured the loading capacity of NPs at different cGAMP: polymer feeding mass ratios (5%, 10%, 15%, and 20%) (Table S2, Supporting Information). The cGAMP: polymer feeding ratio of 20% has not saturated the cGAMP loading and yielded the highest cGAMP loading capacity: 10.2%, 10.6%, and 13.1% for S20, S40, and S60, respectively. Because S40 NPs showed high cGAMP loading capacity, sensitive pH responsiveness (Figure 1C–F), and great biocompatibility (Figure 1I), S40 was selected as the vaccine nanocarriers for further studies. The cGAMP release profile was studied in PB buffer at pH 7.4 and 6.2 (0.1 m), respectively. The pH responsiveness of NPs facilitated cGAMP release in acidic conditions (**Figure** **2**A). To investigate intracellular cGAMP delivery by fluorescence monitoring, we used a fluorescein‐labeled cyclic di‐GMP (Fluo‐CDG) that has similar electrostatic charges, sizes, and chemical structures compared to cGAMP, and is therefore expected to have similar loading and release profile in NPs relative to cGAMP. DC2.4 cells were treated with NP/Fluo‐CDG or free Fluo‐CDG control for 5 h. We observed efficient intracellular delivery of NP/Fluo‐CDG, in contrast to moderate cell uptake of free Fluo‐CDG, by confocal microscopy (Figure 2B and Figure S5, Supporting Information) and flow cytometry (Figure 2D). Note that, despite its negative charge, significant amount of free Fluo‐CDG was delivered to DCs through endolysosome, likely via CDN transporter SLC19A1 on cell surface or fluorescein.^[^ ^27^ ^]^ Nonetheless, NP/Fluo‐CDG showed more efficient intracellular delivery than free Fluo‐CDG. To evaluate endosomal release, we used software ImageJ to analyze the colocalization ratio of Fluo‐CDG with Lysotracker Red‐stained endolysosome. Relative to free Fluo‐CDG, the endolysosome colocalization ratios of NP/Fluo‐CDG, as well as positive controls Lipofectamine 2000 (Lipo/Fluo‐CDG) and PEI (PEI/Fluo‐CDG), were decreased from 1 h post treatment to that at 12 h post treatment, suggesting enhanced endosome escape by NP/Fluo‐CDG (Figure 2C; Figures S6A and S6B, Supporting Information). The ability of these NPs to enhance Fluo‐CDG endosome escape was further confirmed by the reduced endolysosome colocalization with NP/Fluo‐CDG than relative to free Fluo‐CDG, as indicated by the Pearson's *R* correlation values of 0.49, 0.24, 0.25, and 0.34 for free Fluo‐CDG, Lipo/Fluo‐CDG, PEI/Fluo‐CDG, and NP/Fluo‐CDG (Figure S6C, Supporting Information), respectively.^[^ ^28^ ^]^ Of note, part of the Fluo‐CDG presumably escaped from the endolysosome as the NP/Fluo‐CDG complexes, and these Fluo‐CDG nanocomplexes may further protect CDG from cytosolic nuclease degradation and extracellular exportation by CDN exporters such as ectonucleotide pyrophosphatase/phosphodiesterase 1 (ENPP1) in the cytosol.^[^ ^29^ ^]^ Overall, these results demonstrated that NPs efficiently loaded and delivered CDNs into DCs, and facilitated the endosomal escape of CDNs into the cytosol, allowing CDNs to activate cytosolic STING.

We then studied NP/cGAMP for immunostimulation in DC2.4 cells by ELISA and flow cytometry. Compared with free cGAMP, NP/cGAMP efficiently upregulated the expression of maturation marker MHC‐II and costimulatory factors CD40, CD80, and CD86, all of which are critical mediators for antigen presentation and T‐cell priming (Figure 2E; Figures S7 and S8, Supporting Information). Consistently, NP/cGAMP efficiently elicited the secretion of IFN‐I (e.g., IFN‐*α* and IFN‐*β*), and proinflammatory cytokines such as interleukin 12 (IL‐12), in DCs in a dose‐dependent manner (Figure 2F, Figure S9, Supporting Information). Remarkably, the ability of NP/cGAMP to elicit all these cytokines in DCs significantly outperformed that of free cGAMP, cGAMP delivered by Lipofectamine 2000 (Lipo/cGAMP) and PEI (PEI/cGAMP) (Figure 2G), and a state‐of‐the‐art chemically stabilized fluoride‐c‐di‐GMP (F‐CDG) (Figure S9, Supporting Information). To further verify the ability of NP/cGAMP to elicit IFN responses, we used RAW‐Lucia ISG (IFN‐stimulated gene) reporter cells, which are RAW 264.7 murine macrophages with stable integration of an IFN regulatory factor (IRF) inducible Lucia luciferase reporter. As a result, we verified that NP/cGAMP induced IFN responses in a dose‐dependent manner (Figure 2H), and significantly more efficiently than controls of free cGAMP, Lipo/cGAMP, and PEI/cGAMP (Figure 2I). Worth noting, though some pH‐responsive polymers per se can activate STING,^[^ ^26a^ ^]^ our NPs did not show detectable intrinsic STING activation under this experiment condition, suggesting the cGAMP‐selective IFN responses induced by NP/cGAMP (Figure 2I). We used a block polymer PEG‐PDMA to investigate the impact of pH responsive PDPA in S40 on vaccine delivery and immunomodulation. We loaded DY547‐cGAMP in PEG‐PDMA NPs and S40 NPs, and incubated DC2.4 cells with these NVs, respectively, for 18 h. Both NPs mediated efficient DY547‐cGAMP endocytosis into DC2.4 cells (Figure S10A, Supporting Information). The Pearson's *R* value was 0.2 for PEG‐PDMA/cGAMP and 0.08 for S40 NP/cGAMP, indicating decreased endolysosome colocalization with S40 NPs relative to PEG‐PDMA NPs and hence enhanced cGAMP endosome escape by S40 NPs relative to PEG‐PDMA NPs. We also studied cGAMP‐loaded PEG‐PDMA NPs and S40 NPs to induce IFN‐ *β* production in DC2.4 cells by ELISA (Figure S10B, Supporting Information). S40 NPs enabled cGAMP to induce more potent IFN production than PEG‐PDMA NPs. These data suggest that the pH responsiveness of S40 NPs facilitated the endosome escape of NVs and enhanced the ability of cGAMP to elicit IFN‐I responses. Collectively, these data demonstrated the ability of NP/cGAMP to induce potent IFN‐I immunostimulation, providing the basis for them to elicit antitumor innate immunity as well as adaptive immunity when codelivered with antigens in NVs.^[^ ^30^ ^]^

### 2.3 cGAMP/Antigen‐Codelivering NVs Elicited Sustained Antigen Presentation and T‐Cell Responses In Vitro

**Figure 3:**
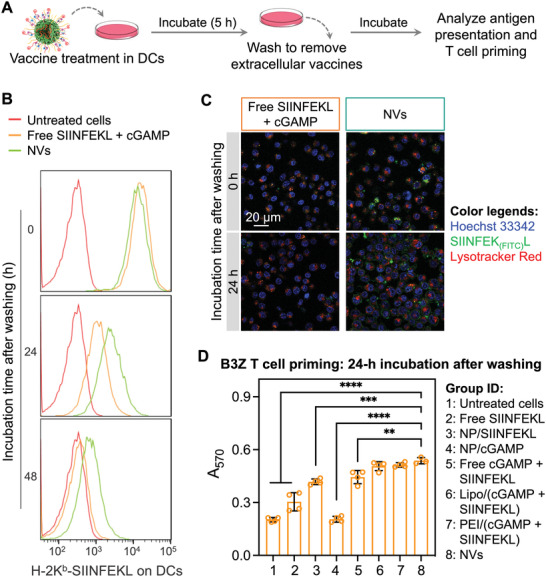
cGAMP/antigen‐codelivering nanovaccines (NVs) sustained antigen presentation in dendritic cells (DCs). A) Study scheme of the antigen presentation sustainability in DC2.4 cells. B) Flow cytometry results showing the levels of SIINFEKL presented on DCs after incubation with NVs [(NP/(cGAMP/SIINFEKL)] and controls for 5 h, washing off extracellular vaccines, and further incubation for a series of durations, prior to antibody staining of cell surface H‐2K^b^‐SIINFEKL complexes. C) Confocal microscopy images showing the uptake and presentation of SIINFEK_(FITC)_L on DCs treated with free vaccines or NVs for 5 h, followed by washing off extracellular vaccines and 24‐h incubation. D) The assay absorption at 570 nm (A_570_) indicated the activity of SIINFEKL‐specific B3Z CD8^+^ T cells after coculture with vaccine‐treated DCs. Relative to controls, NVs enabled DCs to promote antigen‐specific T‐cell activation. Data: mean ± SD (*n =* 3; **p < 0.01, and ****p < 0.0001 (Student's *t*‐test).

Motivated by the potent immunostimulation of NP/cGAMP, we then studied these NPs to codeliver cGAMP and peptide antigens, which are expected to elicit potent adaptive immunity with minimal immune tolerance, the latter caused by the exposure of APCs to peptide antigens in the absence of immunostimulant adjuvants. We first used a model antigen SIINFEKL, an MHC‐I (H‐2K^b^)‐restricted epitope of ovalbumin (OVA). SIINFEKL and cGAMP were efficiently coloaded into NPs as measured by HPLC after ultracentrifugal filtration (Figure S11, Supporting Information). Comparing with S40 NP loaded with cGAMP only, the cGAMP/SIINEKL‐coloaded NVs had high cGAMP loading capacity, likely due to the positive charge of peptides. The resulting NVs showed excellent stability in PBS at room temperature for 5 days (Figure S12, Supporting Information). We then studied antigen presentation of DC2.4 cells treated with the above NP/(cGAMP + SIINFEKL), with the controls of free SIINFEKL, free SIINFEKL + free cGAMP (**Figure** **3**A). After treatment for 5 h, cells were washed and further incubated in fresh medium for a series of durations. The presentation of SIINFEKL by MHC‐I on DCs was stained using a dye‐labeled antibody for the SIINFEKL/H‐2K^b^ complexes (H‐2K^b^ is a subtype of MHC‐I), followed by flow cytometric analysis.^[^ ^25^ ^]^ Prior to washing off at 5 h post treatment, all formulations resulted in efficient antigen presentation on DCs (Figure 3B). Interestingly, after washing off extracellular vaccines at 5 h post treatment, the antigen presentation by cells treated with free vaccines rapidly faded away, likely due to the antigen disassociation from H‐2K^b^ and SIINFEKL degradation. By contrast, NVs sustained the antigen presentation even after washing off extracellular vaccines, likely due to the efficient uptake of NVs that created abundant intracellular vaccine repertoires for sustained vaccine release. This is pivotal because sustained vaccine exposure to immune cells over a prolonged duration potentiates and prolongs adaptive immunity.^[^ ^31^ ^]^ To test this, an FITC‐labeled SIINFEK_(FITC)_L was used to directly analyze antigen presentation by confocal microscopy. Consistently, after incubation for 5 h, strong SIINFEK_(FITC)_L fluorescence was presented on DC2.4 cell surfaces. 24 h after treatment, the SIINFEK_(FITC)_L signal presented on DCs treated with free SIINFEK_(FITC)_L decreased precipitously, in contrast to steady antigen presentation on NV‐treated DCs (Figure 3C and Figure S13, Supporting Information). Note that the antigen presentation shown in Figure 3B was stained using an antibody that binds to MHC‐1/SIINFEKL complexes on the surface of DCs, and the antigen uptake and presentation in Figure 3C was measured by imaging the FITC fluorescence directly from SIINFEK_(FITC)_L. NVs showed higher SIINFEKL signal than free SIINFEKL (Figure 3C), likely because NVs facilitated the cell uptake of SIINFEKL. FITC slightly increased the hydrophobicity of SIINFEKL, which, relative to SIINFEKL, likely enhanced SIINFEK_(FITC)_L loading in NPs, facilitated the cell uptake of SIINFEK_(FITC)_L‐loaded NVs, and may impact the pharmacokinetics of this antigen in vivo. Next, we evaluated the vaccine‐treated DC2.4 cells for T‐cell priming by coculturing the as‐treated DCs with SIINFEKL‐specific B3Z CD8^+^ T‐cell hybridoma. Upon recognition of SIINFEKL/H‐2K^b^ complexes, B3Z cells are activated to produce *β*‐galactosidase, which hydrolyze a substrate into red products that can be quantified by absorbance. As a result, NV‐treated DC2.4 cells enhanced B3Z T‐cell activation relative to control vaccine formulations, including free SIINFEKL, admixed free cGAMP and SIINFEKL, NP/cGAMP, and NP/SIINFEKL. It also showed comparable effect to vaccines delivered by lipofectamine 2000 or PEI that are well known for the suboptimal biocompatibility despite typically good cell transfection efficiency (Figure 3D). Note that some peptides might not be efficiently loaded in lipofectamine, leading to weaker antigen presentation than our NVs. Taken together, these results demonstrated the ability of these pH‐responsive NPs to promote cytosolic vaccine delivery, enhance and prolong sustained antigen presentation, and promote antigen‐specific T‐cell priming by APCs.

### 2.4 Combined ICB and cGAMP/Antigen‐Codelivering NVs Remodeled Tumor Immune Microenvironment for Potent Tumor Immunotherapy

**Figure 4:**
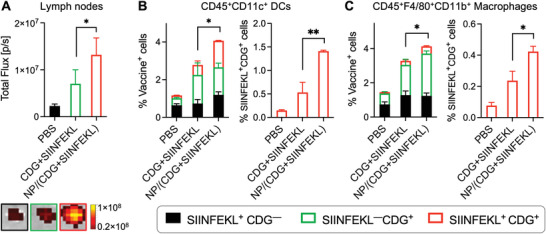
Nanovaccines (NVs) improved the codelivery of DY547‐CDG and SIINFEK_(FITC)_L to draining lymph nodes (LNs) and intranodal antigen presenting cells (APCs). A) Signal quantification (left) and representative photos (right) of draining inguinal LNs 18 h after s.c. administration of NVs or a soluble mixture of SIINFEKL and CDG at tail base in C57BL/6 mice (*n = 3*). B,C) Flow cytometry data quantification showing the NV codelivery of CDG and SIINFEKL into LN‐residing dendritic cells (DCs) (B) and macrophages (C), two primary intranodal APC subsets. Data: mean ± SEM (*n = 3*); *p < 0.05, **p < 0.01, ***p < 0.001, and ****p < 0.0001 (Student's *t*‐test).

Free CDNs and peptide antigens often suffer from poor pharmacokinetics, due to the small sizes and electrostatic charges that lead to random dissemination and rapid clearance from the body. Vaccine delivery systems are thus highly desired to codeliver CDNs and peptide antigens into immunomodulatory tissues and cells, such as LNs and the intranodal APCs, where vaccines can elicit antitumor immunity. To study cGAMP/antigen codelivery by NVs in C57BL/6 mice, we used dye‐labeled DY547‐CDG and SIINFEK_(FITC)_L for fluorescence pharmacokinetic monitoring. A total of 18 h after subcutaneous (s.c.) injection of NP/(CDG + SIINFEKL) and control soluble CDG + SIINFEKL at mouse tail base, draining inguinal LNs were harvested for DY547 fluorescence imaging of CDG. NVs increased CDG accumulation in the LNs, compared with free vaccines (**Figure** **4**A). The quantification of DY547 fluorescence intensities from LNs suggested approximately 5.78‐fold and 1.88‐fold accumulation by NP/(CDG + SIINFEKL) relative to PBS and free CDG + SIINFEKL, respectively (Figure 4A). LNs harbor various immune cells (e.g., APCs, B cells, and T cells) and stromal cells, and have a sophisticated structure that include spacy subcapsular sinus where NVs can reside. Therefore, it is critical to understand the vaccine delivery in intranodal APC subsets that are critical for antigen presentation and antitumor T‐cell responses. Thus, we further dissected the CDG/SIINFEKL codelivery in two main intranodal APC subsets: DCs (CD45^+^CD11c^+^) (Figure 4B) and macrophages (CD45^+^CD11b^+^F4/80^+^) (Figure 4C). As shown by the percentage of SIINFEKL^+^CDG^+^ cells, NVs significantly promoted CDG/SIINFEKL codelivery to DCs and macrophages, relative to free CDG + SIINFEKL (Figure S14, Supporting Information). The efficient vaccine codelivery to LNs and intranodal APCs in vivo provides the basis for NVs to efficiently present antigens from APCs to native T cells and elicit antigen‐specific antitumor T‐cell responses with minimal immune tolerance.

### 2.5 cGAMP/Antigen‐Codelivering NVs Elicited Potent and Durable Antigen‐Specific T‐Cell Responses In Vivo

**Figure 5:**
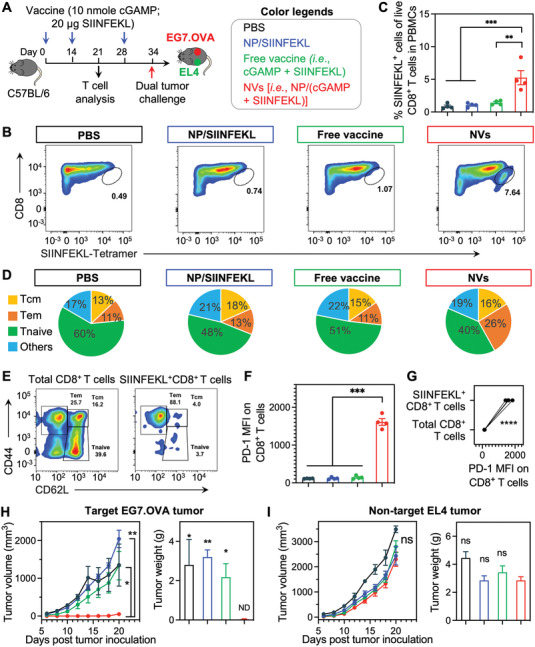
cGAMP/SIINFEKL‐codelivering nanovaccines (NVs) elicited potent and durable T‐cell responses in mice. A) Study design for T‐cell responses in C57Bl/6 mice (*n = 4*). B,C) Representative flow cytometry plots (B) and quantification (C) of a H‐2K^b^‐SIINFEKL tetramer staining assay showing that NVs (s.c. injected at tail base; days 0, 14) augmented the peripheral SIINFEKL‐specific CD8^+^ T cells (day 21) in mice. D) Quantification of CD8^+^ memory T cells in the above immunized mice (day 21). E) Representative flow cytometry plots of memory T cells in total CD8^+^ and SIINFEKL^+^ CD8^+^ T cells in NV‐treated mice (day 21). F) PD‐1 median fluorescence intensity (MFI) on total live PBMC CD8^+^ T cells. G) PD‐1 MFI on total live PBMC CD8^+^ T cells and SIINFEKL^+^ CD8^+^ T cells from NV‐immunized mice, indicating elevated PD‐1 levels specifically on SIINFEKL^+^ CD8^+^ T cells relative to total CD8^+^ T cells. H,I) EG7.OVA (H) and EL4 (I) tumor growth curves and tumor weights at day 20 post tumor challenge in immunized mice challenged with EG7.OVA (right flank) and EL4 (left flank) tumor cells on day 34 post priming vaccination. ND: nondetectable. Statistics are indicated in comparison with NVs. Data represent mean ± SEM; ns: not significant, *p < 0.05, **p < 0.01; ***p < 0.001, and ****p < 0.0001 (one‐way ANOVA with Dunnett test).

Next, we studied the ability of cGAMP/antigen‐codelivering NVs to elicit T‐cell responses in mice. We first studied the MHC‐I‐restricted SIINFEKL as a model antigen that elicits CD8^+^ T‐cell responses. C57Bl/6 mice were s.c. administered (at tail base) with NVs and controls on days 0 and 14 (**Figure** **5**A). On day 21, peripheral blood was collected to analyze the SIINFEKL‐specific CD8^+^ T‐cell response by SIINFEKL/H‐2K^b^ tetramer staining. As shown by flow cytometry, NVs significantly expanded the SIINFEKL‐specific CD8^+^ T cells (Figure 5B,C), with a 6.1‐, 5.0‐, and 3.7‐fold increase relative to PBS, NP/SIINFEKL, and free cGAMP + SIINFEKL, respectively. This demonstrated that NVs elicited potent systemic T‐cell responses. Central memory (T_CM_) and effector memory (T_EM_) cells are two major memory T‐cell subsets. T_CM_ cells access secondary lymphoid organs and proliferate rapidly during secondary immune responses; T_EM_ cells, which are less proliferated and more differentiated than T_CM_ cells,^[^ ^32^ ^]^ preferentially traffic to the peripheral tissues and mediate rapid effector functions of inflammatory reactions or cytotoxicity.^[^ ^33^ ^]^ By staining phenotypical markers CD44 and CD62L on CD8^+^ T cells, we showed that NVs generated large fractions of CD8^+^ T_EM_ cells (CD44^+^CD62L^low^) and CD8^+^ T_CM_ cells (CD44^+^CD62L^high^) (Figure 5D and Figure S16, Supporting Information), including abundant SIINFEKL‐specific CD8^+^ T_EM_ cells (Figure 5E). The ability of NVs to induce immune memory is pivotal to treat metastatic tumor and prevent any tumor recurrence for durable therapeutic response. Meanwhile, NVs upregulated the expression of immune checkpoint PD‐1 on CD8^+^ T cells (Figure 5F and Figure S17, Supporting Information), especially on antigen‐specific CD8^+^ T cells (Figure 5G), indicating the immune exhaustion accompanying potent immunostimulation and providing an opportunity to synergistically combine NVs with anti‐PD‐1 ICB for the optimal therapeutic efficacy. To validate the antigen‐specific T‐cell response and memory, on day 34, the as‐immunized mice were challenged with EG7.OVA on the left flank and control OVA‐negative EL4 cells on the right flank. Consistently, NVs significantly inhibited the growth of target EG7.OVA tumor, but not the nontarget EL4 tumor (Figure 5H,I; Figure S18, Supporting Information). This demonstrates the antigen specificity of NV‐elicited immunity that is critical for effective cancer immunotherapy with minimal nonspecific immunotoxicity or autoimmunity. Collectively, these data demonstrate that cGAMP/antigen‐codelivering NVs elicit potent and durable antigen‐specific T‐cell responses.

### 2.6 Combined ICB and cGAMP/Antigen‐Codelivering NVs Remodeled Tumor Immune Microenvironment for Potent Tumor Immunotherapy

**Figure 6:**
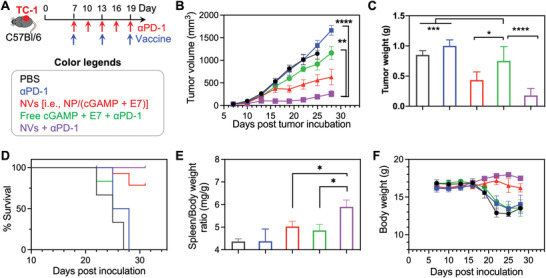
cGAMP/E7‐loaded nanovaccines (NVs) mediated robust immunotherapy in combination with *α*PD‐1 in TC‐1 tumor. A) Study design of TC‐1 combination immunotherapy in syngeneic C57Bl/6 mice. Vaccine: 10 nmole cGAMP and 20 µg antigen, s.c. administration at mouse tail base on days 7, 13, 19; *α*PD‐1: i.p. administration, 200 µg, on days 7, 10, 13, 16, 19. B,C) TC‐1 tumor growth curves (B) and the tumor weights at the end of study on day 28 (C). D) Kaplan–Meier mouse survival curves. E) Spleen/body weight ratios of mice at day 28 after treatment. F) Mouse body weights during the course of treatment. Data represent mean ± SEM; ns: not significant, *p < 0.05, **p < 0.01, ***p < 0.001, and ****p < 0.0001 (one‐way ANOVA with Dunnett test).

Given that these NVs elicited potent and durable T‐cell responses while upregulating PD‐1 expression on CD8^+^ T cells which sensitizes PD‐1 for *α*PD‐1 ICB immunotherapy, we evaluated the therapeutic efficacy of NVs combined with *α*PD‐1. We first studied the immunotherapy of TC‐1 carcinoma expressing human papillomavirus (HPV) oncoviral E7 antigen. HPV infection causes a variety of cancer, including over 90% cervical cancer cases globally. TC‐1 tumor cells were s.c. inoculated in syngeneic C57Bl/6 mice. 7 days later, mice were treated with PBS, *α*PD‐1, NVs [i.e., NP/(cGAMP + E7)], free (cGAMP + E7) + *α*PD‐1, and NVs + *α*PD‐1 (vaccine: 10 nmole cGAMP and 20 µg antigen, s.c. at mouse tail base, 3 times with 6‐day interval; *α*PD‐1: intraperitoneal administration, 200 µg, 5 times with 3‐day interval). NVs significantly inhibited TC‐1 tumor growth, indicating that the adjuvant/antigen codelivery promoted anti‐tumor immunity and immunotherapeutic efficacy. Moreover, NVs + *α*PD‐1 further promoted tumor regression significantly more efficaciously than *α*PD‐1 alone or free vaccine + *α*PD‐1, indicating the therapeutic benefit of these NVs for ICB combination immunotherapy (**Figure** **6**B). These results were verified by the tumor weights at the end of the experiment on day 28 (Figure 6C). Consistently, NVs and NVs + *α*PD‐1 significantly prolonged mouse survival than control treatments (Figure 6D), though the full animal survival profiles were not monitored due to early ending of the experiment. Moreover, the spleen/body weight ratios of mice at day 28 indicate that NVs + *α*PD‐1 promoted splenomegaly likely due to the expansion of proinflammatory immune cells in the spleen (Figure 6E). Splenomegaly has been reported in effective tumor immunotherapy. The splenomegaly on day 28 after tumor inoculation is 9 days after the last vaccine dosing, when free vaccines have been mostly cleared from the body with minimal antitumor immune responses; by contrast, the more durable antitumor immune responses induced by NVs, especially when combined with *α*PD‐1, enhanced splenomegaly relative to controls such as free vaccines. Further, both NVs and the combined NVs with *α*PD‐1 showed great safety signs as indicated by the steady mouse body weights, in contrast to significant body weight loss in mice receiving control treatments. The body weight also dropped for PBS‐treated mice, so we believed the mouse body weight drop was not caused by drug overdosing. We suspected this maybe caused by tumor metastasis and other comorbidity as the tumors progress, which is subject to further investigation. (Figure 6F). Next, in an MC38 colorectal cancer mouse model in syngeneic C57Bl/6 mice, we also evaluated these NVs, alone or combined with *α*PD‐1, for personalized tumor immunotherapy. Specifically, we synthesized NVs as above for the codelivery of cGAMP and a peptide neoantigen Adpgk (ASMTNMELM), which is a MHC‐I‐restricted neoantigen discovered in MC38 cells.^[^ ^34^ ^]^ Again, the combination of NVs [i.e., NP/(cGAMP + Adpgk)] and *α*PD‐1, relative to either NVs or *α*PD‐1 alone, significantly promoted the tumor therapeutic efficacy with great safety (Figure S23, Supporting Information). Though these treatments did not completely control the tumor progression, we envision that their therapeutic efficacy can be improved by approaches such as using multiepitope antigens or synergistic adjuvants. Nonetheless, all these results indicated the synergy between these NVs and ICB: NVs induced anergic antigen‐specific T cells that can be reinvigorated by *α*PD‐1, and *α*PD‐1 potentiated the T‐cell cytotoxicity and prolong T‐cell half‐life for the optimal tumor immunotherapeutic efficacy. Overall, these results demonstrated the great therapeutic efficacy of these NVs, alone or combined with ICB.

**Figure 7:**
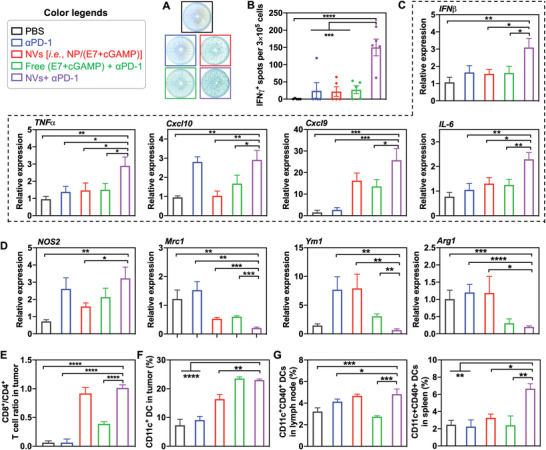
Immune milium analysis in TC‐1 tumor microenvironment after nanovaccines (NVs) and immune checkpoint blockade (ICB) combination immunotherapy. A,B) Photographs (A) and quantification (B) of ELISPOT results showing the INF‐*γ* spots in the as‐treated tumors. C) RT‐PCR results of the mRNA levels of immunostimulatory cytokines and chemokines in as‐treated tumors. D) RT‐PCR results of the mRNA levels of M1‐like macrophage marker *Nos2* and M2‐like macrophage markers *Mrc1, Ym1*, and *Arg1* in as‐treated tumors. Data were relative to the expression of house‐keeping gene *Gapdh*. E) CD8^+^/CD4^+^ T‐cell ratio in TC‐1 tumor after combination immunotherapy. F) Tumor‐infiltrating dendritic cells (DCs) in TC‐1 tumor after combination immunotherapy. G) DC levels in the nontumor draining inguinal lymph nodes (LNs) and spleens of TC‐1‐tumor‐bearing mice after combination immunotherapy. Data represent mean ± SEM; ns: not significant, *p < 0.05, **p < 0.01, ***p < 0.001, and ****p < 0.0001 (one‐way ANOVA with Dunnett test).

The immunosuppressive tumor immune microenvironment (TIME) is where tumor cells and immune cells interact and antitumor immunity is suppressed. Remodeling the TIME to reduce the immunosuppression is pivotal for effective immunotherapy of solid tumors. By ELISPOT analysis of TC‐1 tumor from C57BL/6 mice that were treated as above (Figure 6A), we first studied the ability of our immunotherapy regimens to enhance the secretion of IFN‐*γ*, which is instrumental in the tumor cell killing by antitumor immune cells. We showed that the combination of NP/(cGAMP + E7) with *α*PD‐1 significantly enhanced the IFN‐*γ* secretion in tumor, which is consistent with the enhanced immunotherapeutic efficacy by this combination (**Figure** **7**A,B). We then studied the impact of our immunotherapy on the TC‐1 TIME by RT‐PCR and flow cytometry of the molecular and cellular immune markers. As shown by RT‐PCR, relative to control treatments, NP/(cGAMP + E7) + *α*PD‐1 drastically increased the relative expression of IFN‐I (e.g., *Ifn‐β*), and inflammatory cytokine and chemokine genes such as *Tnf‐α, Cxcl9, Cxcl10*, and *Il‐12* in tumor (Figure 7C). Since *Tnf‐α* and *Il‐6* were also typical M1‐like macrophage biomarkers, we also investigated macrophage polarization by RT‐PCR. Relative to control treatments, NP/(cGAMP + E7) + *α*PD‐1 significantly upregulated representative M1‐like macrophage biomarkers (*Tnf‐α, Il‐6*, and *Nos2*) and downregulated M2‐like macrophage biomarkers (*Mrc1, Ym1*, and *Arg1*) (Figure 7C,D). Further, flow cytometry analysis of the tumor cell milieu (gating tree in Figure S19, Supporting Information) showed that NP/(cGAMP + E7) and NP/(cGAMP + E7) + *α*PD‐1 increased the number of infiltrating CD8^+^ and CD4^+^ T cells (Figure S20A, Supporting Information) and the CD8^+^/CD4^+^ T‐cell ratio (Figure 7E), the latter of which predicts the clinical immunotherapeutic outcome of solid tumors.^[^ ^35^ ^]^ Besides, NP/(cGAMP + E7) + *α*PD‐1 expanded the intratumoral CD11c^+^ DCs and CD11b^+^F4/80^+^ macrophages (Figure 7F; Figure S20B, Supporting Information). These results demonstrated that NP/(cGAMP + E7), especially when combined with ICB, significantly remodeled the TIME to reduce tumor immunosuppression. We further studied the NVs, alone or combined with ICB, for the immunomodulation in secondary lymphoid tissues LNs and spleens. Relative to control treatments, NP/(cGAMP + E7) + *α*PD‐1 increased the expression of costimulatory factor CD40 and CD86 on DCs in LNs (Figure 7G) and spleens (Figure S21, Supporting Information). Moreover, in the draining inguinal LNs of vaccine administration sites (nontumor draining LNs), NP/(cGAMP + E7) + *α*PD‐1 enhanced the frequency of CD11c^+^ DCs, CD11c^+^CD86^+^ DCs, CD11c^+^CD80^+^ DCs, and upregulated CD40 expression on CD11c^+^ DCs (Figure S22, Supporting Information). These results indicate that these treatments activated DCs in LNs where various antitumor immune responses are orchestrated. Overall, these results demonstrate that the combination of NVs and *α*PD‐1 elicited multifaceted antitumor immune responses and reduced the immunosuppression in TIME and secondary lymphoid tissues, all of which are pivotal for effective and durable tumor immunotherapy.

## 3 Conclusion

We designed a pH‐responsive multivesicular polymeric NPs for the codelivery of STING agonist cGAMP and peptide oncoviral antigens and neoantigens to elicit potent and long‐lasting T‐cell responses in combination tumor immunotherapy. The star‐shaped polymers were synthesized based on the Passerini three‐component reaction and ATRP. The hydrophilic and cationic PDMA was synthesized with different chain lengths to optimize cGAMP loading, NV sizes, as well as biocompatibility. The hydrophobic PDPA chain was ultrasensitive pH‐responsive,^[^ ^36^ ^]^ which facilitates NV disassembly in the acidic endolysosome to enhance endosomal escape of cGAMP and antigens. Prolonging PDMA chain increased the cGAMP loading capacity in NPs, but also reduced the NP stability as shown by their high CMC and reduced the pH‐responsiveness as a result of reduced mass fraction of PDPA in the polymer. We chose S40 NPs as the vaccine carriers due to its good safety profile, high loading capacity of cGAMP, and its sensitive pH responsiveness in the pH range (pH 6–7) of endosome, where NVs are expected to be disassembled and escape to cytosol for STING activation by cGAMP and antigen presentation by MHC. In this study, we loaded two peptide antigens into NPs, likely via hydrophobic interactions. Nonetheless, depending on the compositions and sequences, peptides, including tumor antigenic peptides, have vastly heterogeneous physicochemical properties, such as water solubility and electrostatic charges. Such heterogeneity is expected to challenge homogeneous loading of peptide antigens in nanocarriers for wide applications. A previous study has attempted to address this challenge by using a charge‐modified group and a hydrophobic block to tune the NP assembly to a defined size distribution for the loading of peptide antigens with heterogeneous physicochemical properties.^[^ ^18b^ ^]^ NVs efficiently codelivered cGAMP and antigens to draining LNs and key intranodal APC subsets (e.g., DCs and macrophages) in mice. As a result, NVs elicited potent innate immunity in APCs, sustained antigen presentation in a prolonged duration, and promoted vaccine‐stimulated DCs to prime antigen‐specific T cells. Of note, though some pH‐responsive polymers per se have shown to bind and activate STING,^[^ ^26a^ ^]^ we have not observed such intrinsic STING activation ability of our polymers. In mice, NVs elicited potent antigen‐specific CD8^+^ T responses, accompanied with immune memory that is key to treating metastatic tumors and preventing tumor recurrence for durable immunotherapy. The immunostimulation also upregulated the expression of immune checkpoints. Since the low endogenous tumor‐specific effector T cells and the low expression level of immune checkpoints are among the primary causes of tumor resistance to current ICB, the abilities of NVs to expand these cell repertoires and upregulate immune checkpoints are expected to sensitize ICB. This provides an opportunity for rational synergistic combination of NVs and ICB for the optimal tumor immunotherapy. Indeed, relative to free vaccines or ICB alone, NVs, especially combined with ICB, dramatically inhibited tumor progression and prolonged mouse survival in syngeneic TC‐1 and MC38 tumor models. TIME represents a major hurdle for effective tumor immunotherapy. Impressively, NVs, especially combined with ICB, significantly reprogramed the TIME as well as the immune milieu in non‐tumor‐draining LNs and spleens by upregulating immunoactivating cells and molecules and inhibiting immunosuppressive cells. Collectively, these results suggest the great potential of these NVs for adjuvant/antigen codelivery and potent and durable combination cancer immunotherapy.

## Conflict of Interest

The authors declare no conflict of interest.

## Supporting information

Supporting Information

## Data Availability

The data that support the findings of this study are available from the corresponding author upon reasonable request.
