# Supplementary material for: Responsive Multivesicular Polymeric Nanovaccines that Codeliver STING Agonists and Neoantigens for Combination Tumor Immunotherapy
Source: Adv Sci (Weinh). 2022 Jun 16;9(23):2201895. doi: 10.1002/advs.202201895 (PMC9376841; doi:10.1002/advs.202201895)
Supplement: Supplementary file 1 — Supporting Information [file ADVS-9-2201895-s001.pdf]

Supporting Information

pH-responsive multivesicular polymeric nanovaccines that codeliver STING agonists and neoantigens for combination tumor immunotherapy

Ting Su, Furong Cheng, Jialong Qi, Yu Zhang, Shurong Zhou, Lei Mei, Shiwei Fu, Fuwu Zhang, Shuibin Lin *, *Guizhi Zhu* *

Ting Su, Shuibin Lin

Center for Translational Medicine, Precision Medicine Institute, The First Affiliated Hospital, Sun Yat-sen University, Guangzhou 510080, China

Email: linshb6@mail.sysu.edu.cn

Ting Su, Furong Cheng, Jialong Qi, Yu Zhang, Shurong Zhou, Lei Mei, Guizhi Zhu

Department of Pharmaceutics and Center for Pharmaceutical Engineering and Sciences; Institute for Structural Biology and Drug Discovery, School of Pharmacy; The Developmental Therapeutics Program, Massey Cancer Center; Virginia Commonwealth University, Richmond, VA 23298, USA

Guizhi Zhu: gzhu2@vcu.edu

Shiwei Fu, Fuwu Zhang

Department of Chemistry, University of Miami, Coral Gables, FL 33146, United States;

The Dr. John T. Macdonald Foundation Biomedical Nanotechnology Institute, University of Miami, Miami, FL 33136, United States

*** Corresponding authors**

Supplemental Materials and methods

Materials. Poly(ethylene glycol) methyl ether (Mw=2000 g/mol) (mPEG2K), 2-(N,N-Diisopropylamino)ethyl methacrylate, 2-(Dimethylamino)ethyl methacrylate, 3-Azido-1-propanol, N,N,N',N'',N''-Pentamethyldiethylenetriamine (PMDETA), 2-Bromoisobutanoyl bromide, Triethylamine (TEA) and CuBr were purchased from Sigma-Aldrich. CuBr was stirred in glacial acetic acid for several times until the solution become colorless, then washed by acetone and dried under vacuum at room temperature. Monomers of 2-(dimethylamino)ethyl2-(N,N-Diisopropylamino)ethyl methacrylate and 2-(Dimethylamino)ethyl methacrylate were passed through passed through an alumina column before use to remove the hydroquinone inhibitors. Other solvents and reagents were used as received. cGAMP was purchased from InvivoGen. Fluorescein-labeled cyclic di-GMP was purchased from BioLog Life Science Institute.

Synthesis of the star-shape polymer PEG(-g-PDMA)-b-PDPA. The synthesis route of PEG(-*g*-PDMA)-*b*-PDPA was showed in **Figure S1**. First, we synthesized a dual end-functionalized PEG(-alkynyl)-Br *via* Passerini three-component reaction as reported before[24]. PEG(-alkynyl)-*b*-PDPA was synthesized by atom-transfer radical-polymerization (ATRP). A mixture of PEG(-alkynyl)-Br (0.500 g), 2-(N,N-Diisopropylamino)ethyl methacrylate (1.073 g), and (PMDETA) (0.433 g) was dissolved in THF. After three times of freeze-pump-thaw cycles, CuBr (0.361 g) was introduced quickly into this reaction mixture under nitrogen atmosphere. The mixture was placed in an oil bath at 40 °C overnight. The polymerization was quenched by adding excessive THF and stirring in exposure to air. The solution was passed through an Al_2O_3 column and evaporated to dryness under vacuum. The residue was diluted with THF and precipitated in excess of diethyl ether three times. PEG(-alkynyl)-*b*-PDPA was obtained after vacuum drying.

A small molecule atom transfer radical polymerization (ATRP) initiator 3-Azidopropyl 2-bromoisobutyrate was synthesized. 3-Azido-1-propanol (0.300 g) and 2-Bromoisobutanoyl bromide (0.820 g) were dissolved in anhydrous dichloromethane under nitrogen atmosphere and put into an ice base. TEA (0.360 g) dissolved in anhydrous dichloromethane was slowly dropped into the mixture and reacted overnight at 0 °C. Next, the suspension was filtered, and the filtrate was washed with saturated $NaHCO_3$ aqueous solution three times and saturated NaCl for once. The product was dried over $MgSO_4$ and concentrated under vacuum. The crude product was purified by column chromatography on silica using heptane/ethyl acetate (9:1) mixture as the eluent. The final ATRP initiator, 3-azidopropyl 2-bromoisobutyrate (AEBiB), was obtained as a slightly yellow oil. A mixture of AEBiB (0.159), 2-(Dimethylamino)ethyl methacrylate (2.00 g), and PMDETA (1.10 g) were dissolved in THF. After three times of freeze-pump-thaw cycles, CuBr (0.914 g) was introduced quickly into this reaction mixture under nitrogen atmosphere. The ATRP reaction was performed as described above, and PDMA-N_3 was obtained as a viscous polymer. Different feeding ratios of monomers to the initiator (20, 40, 60) were designed.

We synthesized the final product by click chemistry. PEG(-alkynyl)-*b*-PDPA (0.827 g), PDMA-N_3 (0.300 g), and PMDETA (0.276 g) were dissolved in anhydrous DMF. After three times freeze-pump-thaw cycle, CuBr (0.228 g) was added quickly into the mixture under the protection of nitrogen. The click reaction was carried out at 60 °C. After 24 h, the reaction was diluted with THF and stirred in the air to stop the reaction. The mixture was passed through an Al_2O_3 column to remove the copper catalyst. Then the solution was dialyzed against deionized water for three days and was lyophilized to obtain the final product PEG(-*g*-PDMA)-*b*-PDPA.

Micelle preparation and characterization. Blank micelles were prepared by a cosolvent evaporation method. In brief, 5 mg polymer was dissolved in THF; then the organic phase was dropped into 5 mL DI water under magnetic stir until THF was fully evaporated. Subsequently, the solutions were filtered by 0.45 µM filters to remove the undissolved substance. The sizes and morphology of the micelles were characterized by dynamic light scattering (DLS) and transmission electron microscopy (TEM, Jeol JEM-1400Plus).

The critical micelle concentration (CMC) of these polymers was investigated by using pyrene as a hydrophobic fluorescence probe. Polymeric NP solution of different concentrations with the same pyrene concentration of 6×10^{-7} M was prepared. Fluorescence spectra of pyrene were recorded with a fluorescence spectrophotometer (PTI QuantaMaster, HORIBA). The emission fluorescence intensities at 331.4 and 334.2 nm were monitored, and the intensity ratios of I334.2/I331.5 were recorded.

pH titration of polymers was carried out to evaluate their buffering capacities. In a typical procedure, 1 mg/mL micelle solution was prepared, and the pH was adjusted initially to 3 by adding 0.1 M HCl. Then, pH titration was carried out by adding small volumes (10 µL increments) of 0.01 M NaOH solution under stirring. The pH increases in the range of 3 to 10 were monitored by a pH meter with a microelectrode.

pH-responsiveness of the polymeric micelles. pH-responsiveness of the polymeric micelles were studied by measuring the changes of hydrodynamic sizes and zeta potential under different pH. Briefly, 0.1 mL of micelle solution (1 mg/mL) was mixed thoroughly with 0.9 mL phosphate buffer (PB) solutions (0.1 M) at different pH ranging from 5.0 to 7.4. After incubation overnight at room temperature, the sizes and zeta potential were measured using a zeta sizer.

Red blood cell (erythrocyte)hemolysis assay at different pH was also used to investigate the pH responsiveness. Erythrocytes collected from mice were pelleted at 1,000 rpm for 3 min and washed three times with PBS. Following the third wash, the erythrocytes were suspended in micelle solution in 0.1 M PB buffer at desired pH ($n = 3$). The resulting erythrocytes were incubated at 37 °C for 1 h, then centrifuged at 1,000 rpm for 5 min, and the supernatant was transferred to a 96-well plate to quantify the hemoglobin leakage *via* absorbance spectrometry (λ = 575 nm).

cGAMP loading and release. cGAMP loaded polymer NPs was prepared by adding cGAMP into the polymer solution and stirring for 0.5 h. Then the cGAMP-loaded NPs were purified by ultrafiltration (centrifugal filters,10 kDa NMWCO, Millipore, USA) at 10,000 g for 10 min. The filtrate was collected, and nuclease-free water was added and repeated ultrafiltration to wash off unloaded cGAMP. Next, all the filtrate was collected for high performance liquid chromatography (HPLC) analysis using the mobile phase of 90% water with 0.1% trifluoroacetic acid and 10% acetonitrile with 0.1% trifluoroacetic acid to determine the cGAMP concentration. The loading capacity and loading efficiency were calculated by the following equations:

Loading capacity = (loaded cGAMP weight)/(polymer weight+ loaded cGAMP weight);

Loading efficiency = (total cGAMP - washed cGAMP)/(total cGAMP).

For *in vitro* cGAMP release studies, purified cGAMP-loaded NPs were added into 10,000 MWCO Slide-A-Lyzer MINI Dialysis units (Thermo Fisher). The units were dialyzed against PBS of different pH values at 37 °C. At each time point, 0.1 mL PBS containing the released cGAMP was taken out for HPLC measurement, and the same volume of fresh PBS was added to the dialysate.

Preparation of peptides loaded NVs. The peptides (SIINFKEL and Adpgk) were dissolved in water at low concentrations. We first prepared S40 blank micelles by cosolvent evaporation, and evaporate the organic phase. Then the peptide and cGAMP solutions were dropped slowly into the micelle solution under magnetic stirring. Next, the mixed solution was subject to ultracentrifugal filtration (10 kDa NMWCO, Millipore, USA) to remove the unloaded peptides and cGAMP. The loading capacity was determined by HPLC with the mobile phase of 35% water with 0.1% trifluoroacetic acid (Buffer A) and 65% acetonitrile with 0.1% trifluoroacetic acid (Buffer B) for SIINFEKL. For Adpgk, the mobile phase was gradient elution by Buffer B from 30% to 60% in 20 minutes. The cGAMP elution method was the same as used in cGAMP loading. The stability of the NVs under physiological conditions was measured in PBS at 37 °C for 5 days.

Cells. DC2.4 cells and TC-1 cells were cultured in RPMI 1640 medium. RAW-ISG cells were obtained from InvivoGen and cultured in DMEM medium with 100 μg/mL Normocin and 200 μg/mL Zeocin. Murine colon adenocarcinoma MC38 cells were cultured in DMEM medium. All medium was supplemented with 10% FBS and 0.1% penicillin and streptomycin. B3Z cells were cultured with RPMI 1640 medium supplemented with 10% FBS, 0.1% penicillin and streptomycin, 2 mM L-glutamine, 1 mM sodium pyruvate, and 50 μM 2-mercaptoethanol. All cells were cultured in a humidified atmosphere (5% CO_2, 37 °C) in a Biosafety Level II cabinet.

Evaluation of antigen presentation, activation, and cross-priming. *In vitro* antigen presentation was evaluated in DC2.4 cells through ELISA and flow cytometry. Specifically, cells were seeded in 96-well plates at a density of 3,000 cells per well. Then cells were treated with different cGAMP formulations for 24 h. Then, supernatants were collected for cytokine detection by ELISA Kit (mIFN-α from InvivoGen; mIFN-β and mIL-12p70 from R&D Systems) following the manufacturer's instructions. RAW-ISG cells were seeded in 96-well plate (0.03×10^6 cells/well) and cultured overnight. cGAMP formulations were added and cultured for 24 h. Then 50 μL supernatants medium and 50 μL Quanti-Luc assay solution (InvivoGen) were mixed together into a 96-well white opaque plate for IFN signaling stimulation. The expression levels of costimulatory factors on DC2.4 cells were measured by flow cytometry. Cells were seeded into 6-well plates at a density of 10,000 cells per well. Then cells were treated with medium containing different cGAMP formulations for 24 h. After incubation, cells were harvested, washed twice, and resuspended in PBS for antibody staining for 0.5 h on ice (PE anti-mouse I-A/I-E, PerCP anti-mouse CD86, Alexa Fluor647 anti-mouse CD80, and FITC-anti mouse CD40, BioLegend). Cells were then washed with PBS for three times for flow cytometry using a CytoFLEX Flow Cytometer from Beckman Coulter Life Sciences.

In vitro cell uptake. In vitro cell uptake was studied by confocal laser scanning microscopy (CLSM) and flow cytometry. For CLSM, DC2.4 cells were cultured on glass dishes at a cell density of 1×10^4 cells per mL. Then, free fluorescein-modified cyclic di-GMP (Fluo-CDG) or polymeric loaded Fluo-CDG were added to cells. After incubation for different time points, cells were stained with Lysotracker Red (Life Technologies) and Hoechst 33342 (Life Technologies) according to the manufacturers' instructions. Cells were imaged on a Zeiss LSM 710 confocal microscope. For flow cytometry, DC2.4 cells were seeded on

6-well plates, and then treated with Fluo-CDG just as in the CLSM experiment. After different time points, cells were harvested, washed twice for flow cytometry using CytoFLEX Flow Cytometer from Beckman Coulter Life Sciences.

In vitro evaluation of antigen cross-priming. To evaluate cross-presentation of antigens from DC2.4 cells to T cells, CLSM was firstly used to observe the antigen presentation properties on the surface of DC2.4 cells. We used a fluorescein isothiocyanate (FITC) modified-model antigen SIINFEK$_{(FITC)}$L, which maintained the binding ability of SIINFEKL to the H-2K^b molecules.[25] NP/(cGAMP + SIINFEK$_{(FITC)}$L) or soluble SIINFEK$_{(FITC)}$L was incubated with DC2.4 cells on glass-bottom dishes. After incubation for 5 h, the medium was replaced with fresh medium and further incubated for different time points. Then DC2.4 cells were stained with Hoechst 33342 and observed by confocal microscopy. Flow cytometry experiment was also performed for antigen presentation studies. DC2.4 cells were seeded on 6-well plates and treated with NP/(cGAMP + SIINFEKL) or soluble cGAMP + SIINFEKL. After incubation, DC2.4 cells were collected and stained with APC-labeled anti-SIINFEKL/H-2K^b antibody (BioLegend) for 0.5 hours on ice and then analyzed by flow cytometer.

To evaluate antigen cross-priming, a cell co-culture model comprised of DC2.4 cells and B3Z T cell hybridoma was performed. B3Z cells are SIINFEKL-specific CD8$^+$ T-cell hybridoma, which can be activated to produce β-galactosidase (β-gal) upon recognition of H-2K^b/SIINFEKL complex. The β-gal can hydrolyze the substrate of chlorophenol red-β-D-galactopyranoside (CPRG) into red products. DC2.4 cells were seeded into 96-well plates and treated with different SIINFEKL formulations. After incubation, medium was aspirated, and DC2.4 cells were washed, then co-cultured with 10^4 B3Z cells for another 24 h. Then cells were lysed for 4 h at 37 °C with lysis buffer (PBS with 100 mM 2-mercaptoethanol, 9 mM $MgCl_2$, 0.2% Triton X-100 and 0.15 mM CPRG). The reaction was stopped by 1 M sodium carbonate. The magnitude of antigen priming was evaluated through absorbance measurements (λ = 570 nm).

Animals. All animal work was conducted following NIH guidelines and in accordance with an approved protocol by the Virginia Commonwealth University Animal Care and Use Committee (IACUC). Female C57BL/6 mice (6-8 weeks) were purchased from the Charles Rivers.

In vivo NV delivery to draining LNs and intranodal cells. To evaluate LN delivery, DY547-modified c-diGMP and FITC-modified SIINFEKL were used. Balb/c mice were subcutaneously injected with different formulations at the tail base. After 18 h, draining inguinal LNs were harvested, and the DY547 signal was measured by IVIS (Caliper Life Sciences). In another cohort, LNs were treated with collagenase D (1 mg/mL, Sigma) and DNase I (10 U/mL, Sigma) for 0.5 h at 37 °C. Then the LNs were dissociated, and single cell suspensions were prepared. The resulting single cells were stained with Zombie Aqua for live/dead and then stained with CD45-BV421, CD11c-APC for DCs, and CD11b-PE/CY5, F4/80-APC/Cy7 for macrophages. Cells were then washed, fixed, and analyzed for DY547 and FITC fluorescence signals in DCs and macrophages on a BD LSR Fortessa flow cytometer. Representative flow cytometry gating trees are shown in **Figure S19**.

In vivo immunization and antigen-specific T cell response. SIINFEKL was used as a model antigen to study *in vivo* T cell responses. C57BL/6 mice were immunized *via* subcutaneous injection at the base of the tail with different formulations of vaccines on days 0 and 14: (1) PBS, (2) NP/SIINFEKL, (3) NP/(cGAMP + SIINFEKL), and (4) Free cGAMP + Free SIINFEKL. On day 21, peripheral blood cells were collected, and red blood cells were lysed using ACK lysis buffer (Thermo Fisher Scientific) for 5 min, and then the blood clot was removed by centrifugation. Cells were washed twice in PBS and stained using Zombie Aqua (Biolegend). Then cells were suspended in cold PBS supplemented with 2% FBS and stained with dye-labeled staining cocktail including CD8α-APC/Cy7, CD44-Alexa Fluor 647, CD62L-FITC, PD-1-Brilliant Violet 421 (BioLegend), and PE-Tetramer H-2K^b-restricted SIINFEKL (NIH Tetramer Core Facility). Then

cells were washed, resuspended in Cytofix (BioLegend) for 20 min at 4°C. After fixing, cells were washed with Perm/Wash buffer twice and resuspended for flow cytometry quantification using a BD LSRFortessa-X20.

On day 28, mice were vaccinated with different formulations of SIINFEKL again. On day 34, tumor challenge was conducted by subcutaneously inoculation with OVA-positive tumor cells EG7.OVA (0.8 × 10^6) on the right shoulder and control OVA-negative lymphoblastoma EL4 (0.8 × 10^6) on the left shoulder. Tumor sizes and body weight were monitored every 3 days. Mice were euthanized if tumor volume exceeded 2,000 mm^3. Tumor volume was calculated as Volume = (length × width × with)/2.

In vivo tumor models and combination cancer immunotherapy. Female C57BL/6 mice (6-8 weeks) were subcutaneously injected with MC38 cells (3 × 10^6) on the right shoulder. On day 6, mice were randomly divided into 7 groups (n = 6-7) and vaccinated with (1) PBS, (2) αPD-1, (3) NP/(cGAMP + Adpgk), (4) NP/(cGAMP) + αPD-1, (5) NP/(Adpgk) + αPD-1, (6) NP/(cGAMP + Adpgk)+ αPD-1, (7) free (cGAMP + Adpgk) + αPD-1. Vaccines consisting of antigen peptides (20 µg per mouse) and cGAMP (10 nmol per mouse) were injected subcutaneously at the tail base on days 6, 12, and 18. For combination immunotherapy, mouse αPD-1 (200 µg per mouse) were administered intraperitoneally on days 6, 9, 12, 15, and 18. Tumor volumes and body weights were monitored every 3 days. Mice were euthanized if the tumor volume exceeded 2,000 mm3. Tumor volume was calculated as (length × width × with)/2.

In TC-1 tumor model, female C57BL/6 mice (6-8 weeks) were subcutaneously injected with TC-1 cells (1 × 10^5) on the right shoulder. On day 6, mice were randomly divided into 7 groups (*n = 6-7*) and vaccinated with (1) PBS, (2) αPD-1, (3) NP/(cGAMP + E7), (4) NP/(cGAMP + E7) + αPD-1, (5) free (cGAMP + E7) + αPD-1. Vaccines consisting of antigen peptides (20 µg per mouse) and cGAMP (10 nmol per mouse) were injected subcutaneously at the tail base on days 7, 13, and 19. Again, mouse αPD-1 (200 µg per mouse) were administered intraperitoneally on days 7, 10, 13, 16, and 19, followed by monitoring tumor volume and body weight monitoring as above. On day 30, mice were sacrificed, and tumors, spleens, and LNs were collected for the following immune analysis.

Enzyme-linked immunospot (ELISPOT). TC-1 tumor-bearing mice were sacrificed on day 30 post tumor inoculation to excise spleens, followed by grinding spleen tissues and lysing and removing red blood cells. 0.3 × 10^6 splenocytes/well were plated in RPMI 1640 media in a 96-well ELISPOT plate coated with anti-IFNγ (BD Biosciences, 551083). E7 peptide (5 µg/mL) was added and incubated with splenocytes for 18 hours at 37 °C. Plates were then processed and read on a Bioreader 7000 Elispot plate reader (MITS Core, University of Virginia).

Gene expression in the tumor microenvironment. Tumor RNA was isolated with an RNA extraction kit (Invitrogen) according to the manufacturer's specifications. Complementary DNA (cDNA) was synthesized with a High-Capacity cDNA Reverse Transcription Kit (Thermo Fisher Scientific). The gene expression was analyzed *via* Quantitative real-time PCR (qPCR) using the appropriate SYBR™ Green PCR Master Mix (Thermo Fisher Scientific). Primer sequences are shown in supplemental **Table 2**.

Flow cytometric analysis of tumor macroenvironment, spleen, and LNs. Tissues of interest were treated with collagenase D (1 mg/mL, Sigma) and DNase I (10 U/mL, Sigma) for 2 h at 37 °C. Then the tumors were dissociated and passed through a 70 mm strainer to prepare single cells. The resulting single cells were stained with Zombie Aqua for live/dead and the following antibodies: CD45-PE, CD3-Alexa Fluor 488, CD4-PerCP/CY5, CD8α-APC/CY7, and CD11c-Alexa Fluor 594 (BioLegend). Samples were run on BD LSRFortessa-X20 and analyzed in FlowJo to identify T cells ($CD45^+CD3^+CD4^+$)/($CD45^+CD3^+CD8\alpha^+$) and DCs ($CD45^+CD11c^+$). Representative flow cytometry gating trees are shown in **Figure S19**.

Supplemental Figures

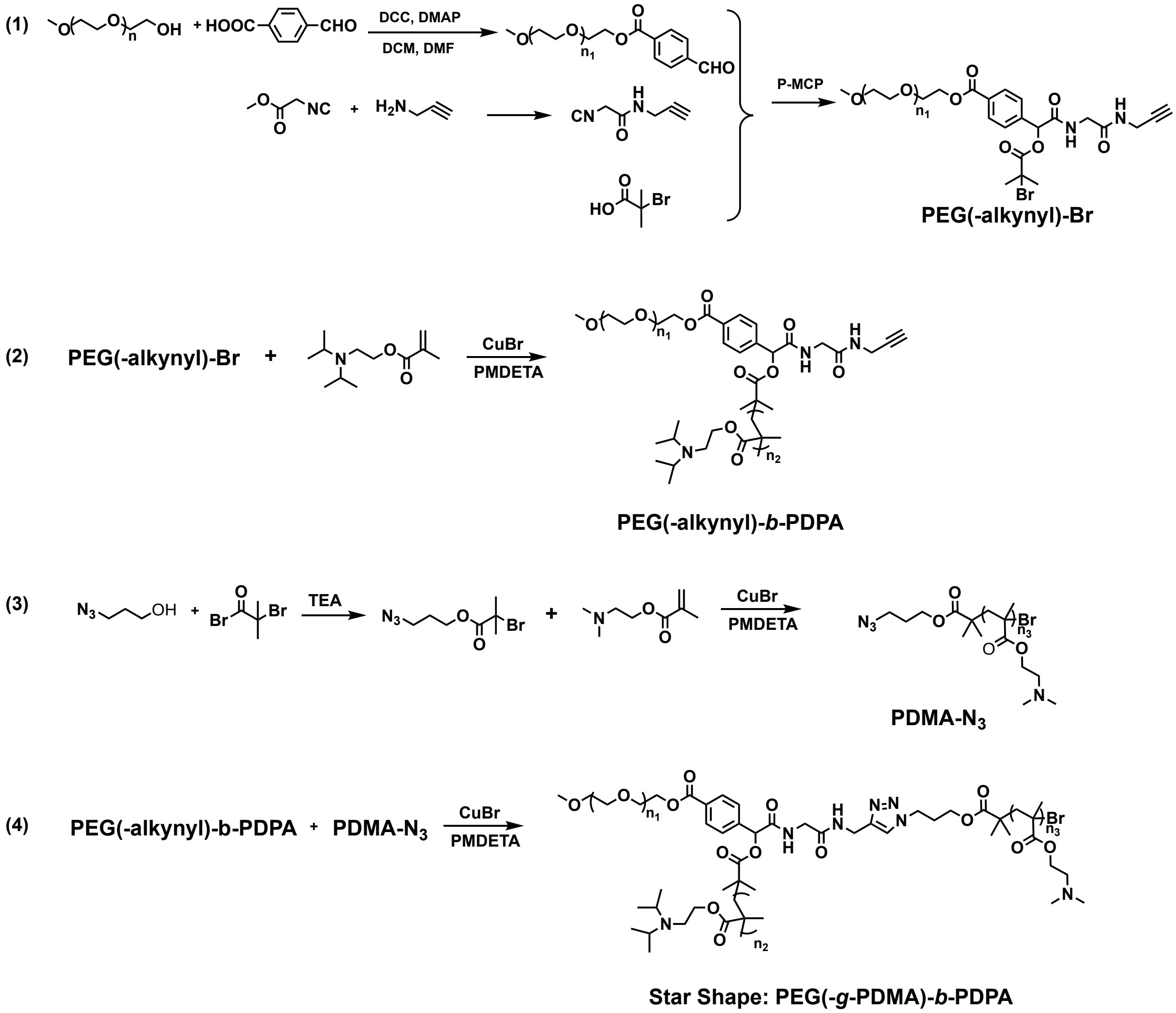

Figure S1. The synthetic routes of the pH responsive polymers.

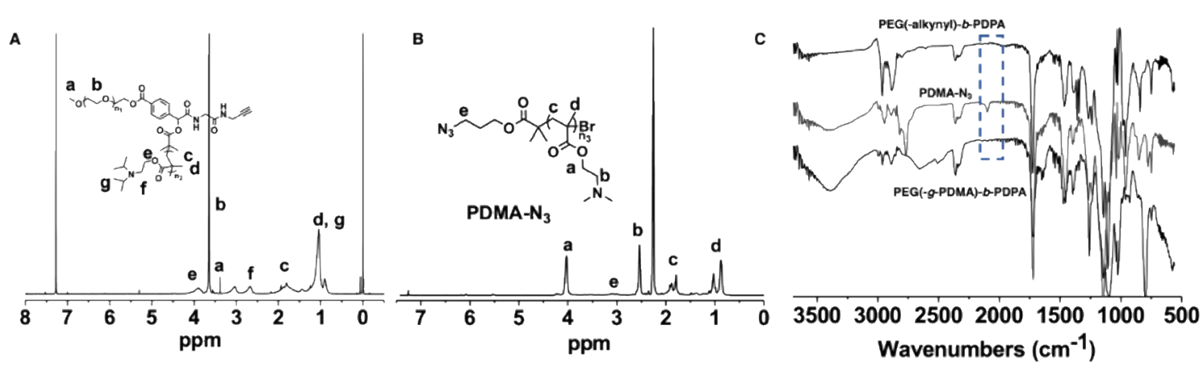

Figure S2. ^{1}H NMR spectra and the Fourier transform infrared (FTIR) spectra of the polymers. (A) ^{1}H NMR spectrum of PEG(-alkynyl)-*b*-PDPA in $CDCl_3$ (400 MHz). (B) ^{1}H NMR spectrum of PDMA-N_3 in $CDCl_3$ (400 MHz). (C) Fourier transform infrared (FTIR) spectra of representative PEG(-alkynyl)-*b*-PDPA, PDMA-N_3, and PEG(-*g*-PDMA)-*b*-PDPA. The characteristic peak at 2100 cm^{-1} from azide stretching vibration disappeared after the click reaction, suggesting the polymer was successfully synthesized.

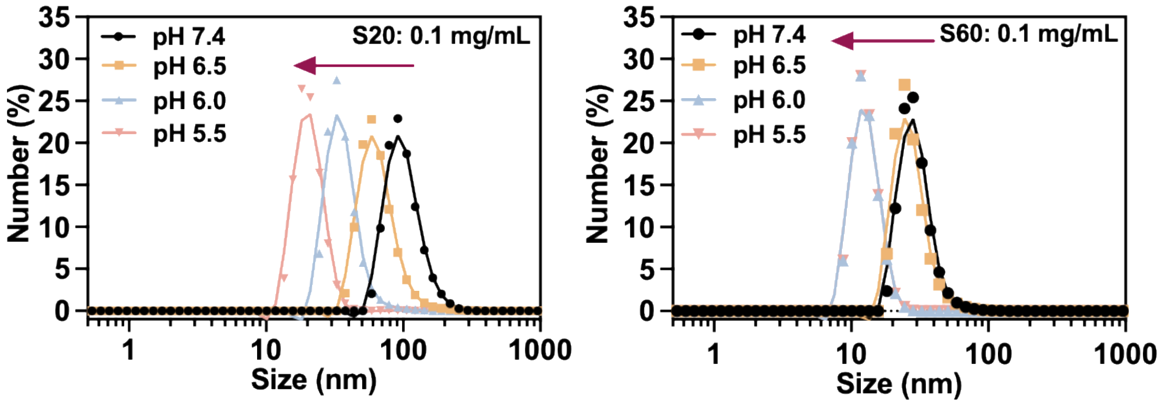

Figure S3. DLS results showing the hydrodynamic sizes of S20 and S60 NPs (1 mg/mL) at different pH conditions, indicating the pH-responsive NP disassembly in acidic conditions.

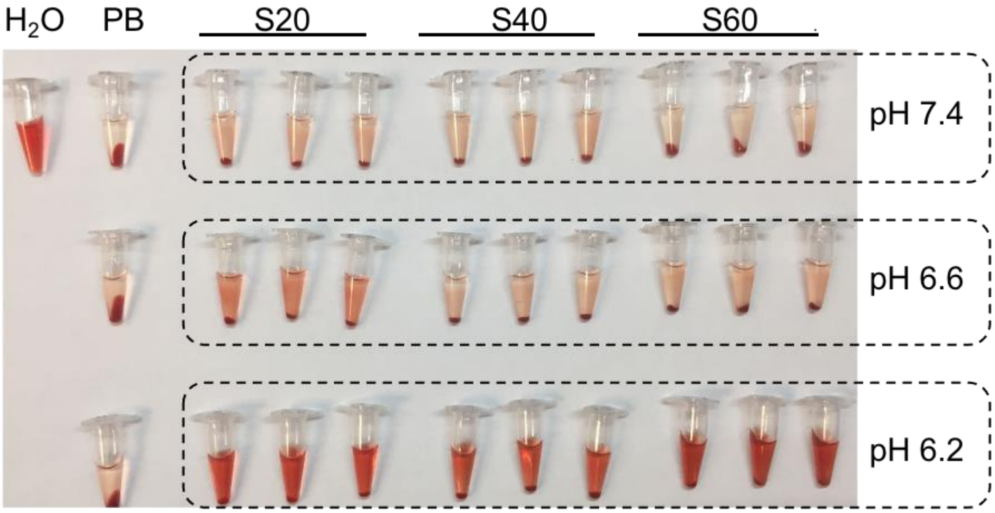

Figure S4. The photograph of hemolysis analysis for three polymers S20, S40, and S60 at different pH.

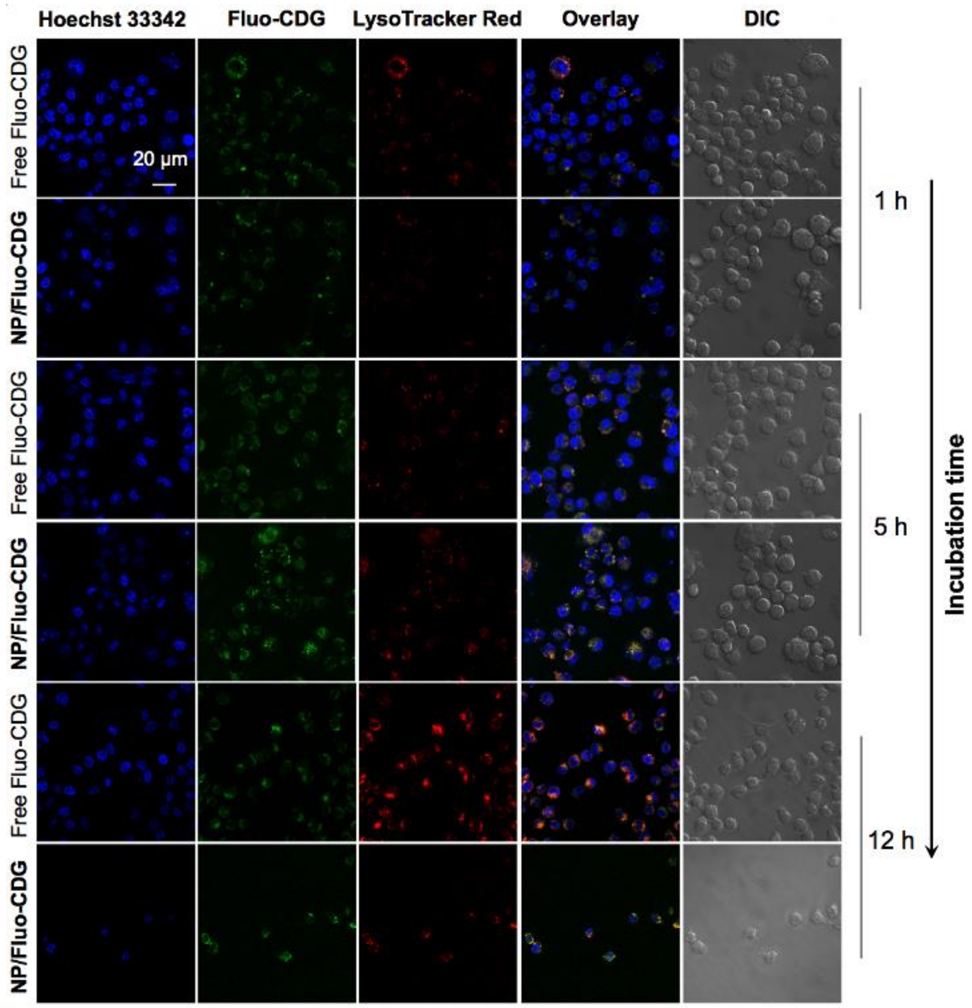

Figure S5. Confocal microscopy images showing the cellular uptake of Fluo-CDG-loaded S40 NPs (NP/Fluo-CDG) compared with free Fluo-CDG in DC2.4 cells. The time noted on the right is the incubation time.

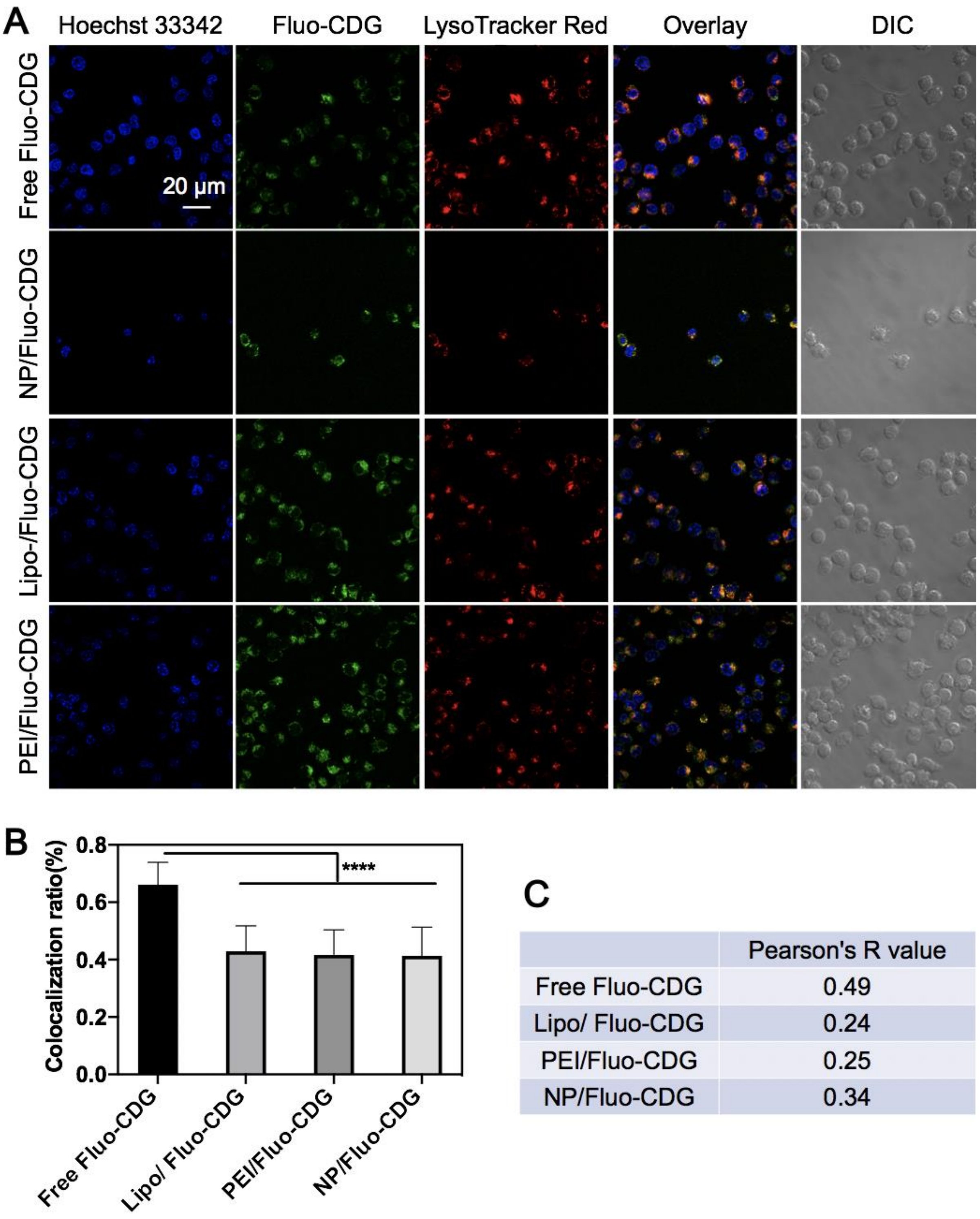

	Pearson's R value
Free Fluo-CDG	0.49
Lipo/ Fluo-CDG	0.24
PEI/Fluo-CDG	0.25
NP/Fluo-CDG	0.34

Figure S6. NVs promoted the endosome escape of STING agonist CDG. (A) Confocal microscopy images showing the colocalization ratio of Fluo-CDG with endolysosomes after 12 h incubation in DC2.4 cells treated with different formulations: free Fluo-CDG, S40 loaded with Fluo-CDG (NPs/Fluo-CDG),

Lipofectamine 2000 loaded with Fluo-CDG (Lipo/ Fluo-CDG), and PEI mixed with Fluo-CDG (PEI/Fluo-CDG). DIC: differential interference contrast. (B) The colocalization ratio of Fluo-CDG with lysosomes by their intensity by analysis with ImageJ. (C) The Pearson's R correlation value calculated through ImageJ.

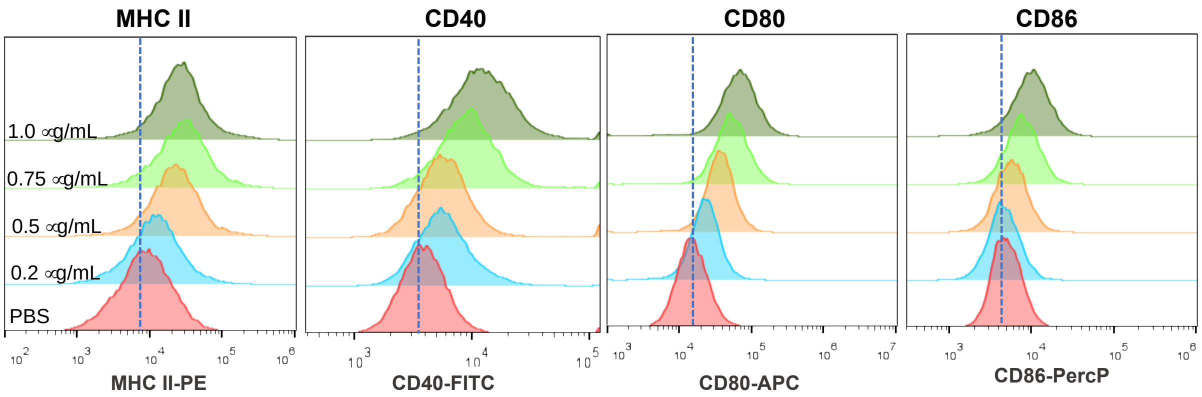

Figure S7. Dose-dependent expression of MHC-II, and co-stimulatory factors CD40, CD80, and CD86 on DC2.4 cells after incubation with S40-NPs/cGAMP.

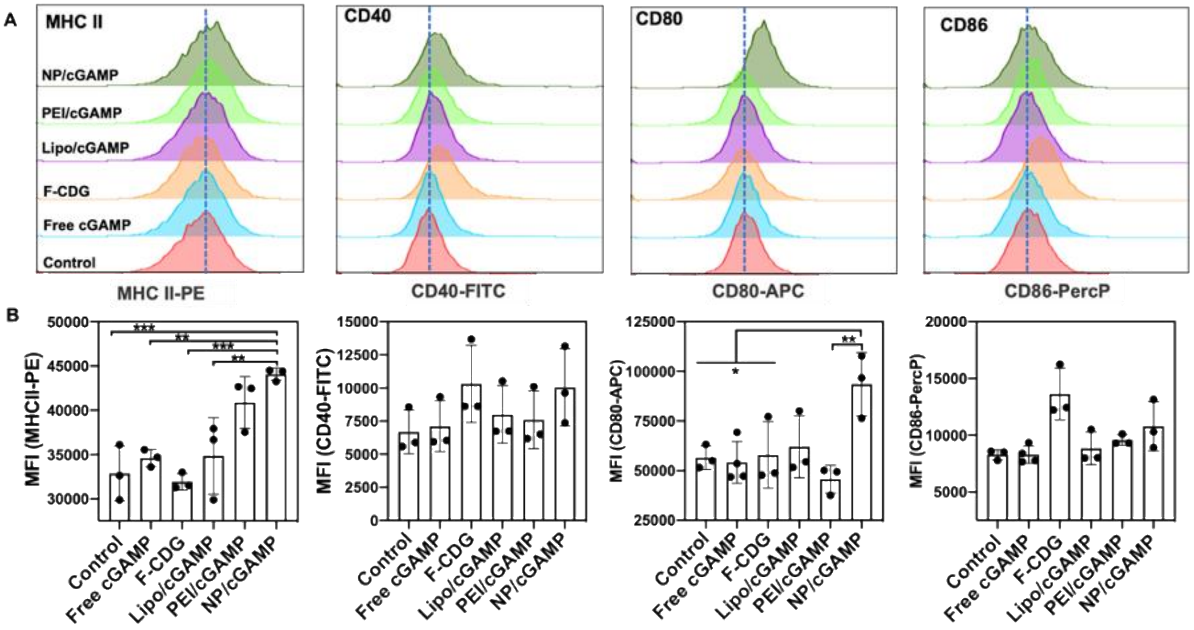

Figure S8. (A) The expression of MHCII, and co-stimulatory factors CD40, CD80, and CD86 on DC2.4 cells after incubation with different formulations of cGAMP. F-CDG: fluoride-modified CDG as a control of nuclease-resistant CDG. PEI/cGAMP: PEI mixed with cGAMP. NP/cGAMP: S40 loaded with cGAMP. (B) Median fluorescence intensity (MFI) of MHCII, CD40, CD80, and CD86 on DC2.4 cells after incubation with different formulations of cGAMP. The concentration of cGAMP was 1 μg/mL.

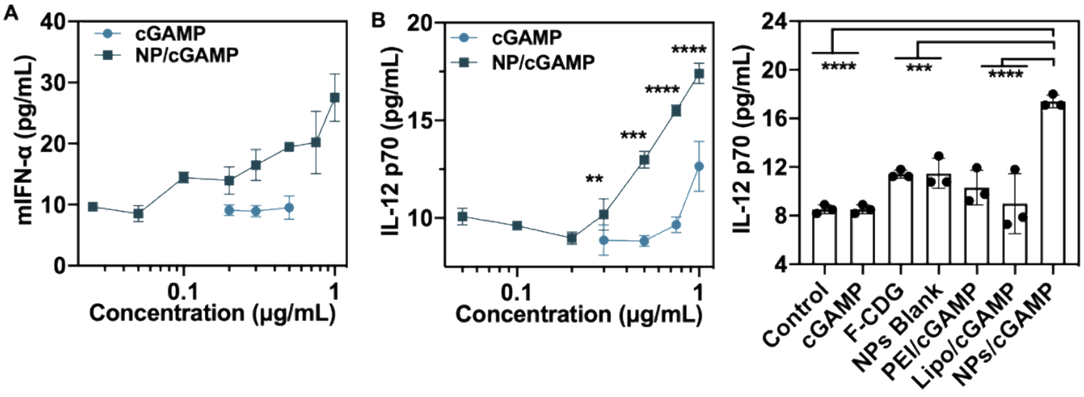

Figure S9. ELISA results showing that S40 NPs significantly promoted the ability of cGAMP to elicit the production of mouse IFN-α (A) and IL-12p70 (B) in DC2.4 cells after a 24-h incubation (cGAMP: 1 μg/mL).

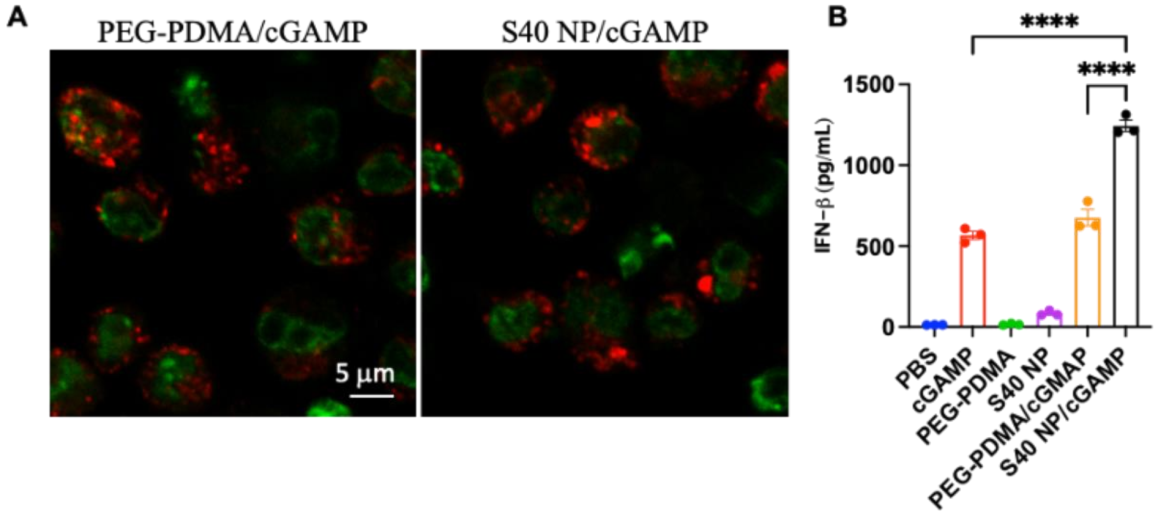

Figure S10. (A) Confocal microscopy images showing the colocalization of DY547-cGAMP with endolysosomes after 18-h incubation in DC2.4 cells by PEG-PDMA/cGAMP and S40 NP/cGAMP (Red: DY547-cGAMP; Green: lysotracker green). As calculated using ImageJ software, the Pearson's R values were 0.2 for PEG-PDMA/cGAMP and 0.08 for S40 NP/cGAMP, indicating more efficient cGAMP endosome escape by S40 NPs. This suggest that the pH responsiveness of S40 NPs likely facilitated the endosome escape of NVs. (B) ELISA results showing that relative to PEG-PDMA NPs, S40 NPs significantly promoted the ability of cGAMP to produce mouse IFN-β in DC2.4 cells after a 24-h incubation (cGAMP: 10 μg/mL).

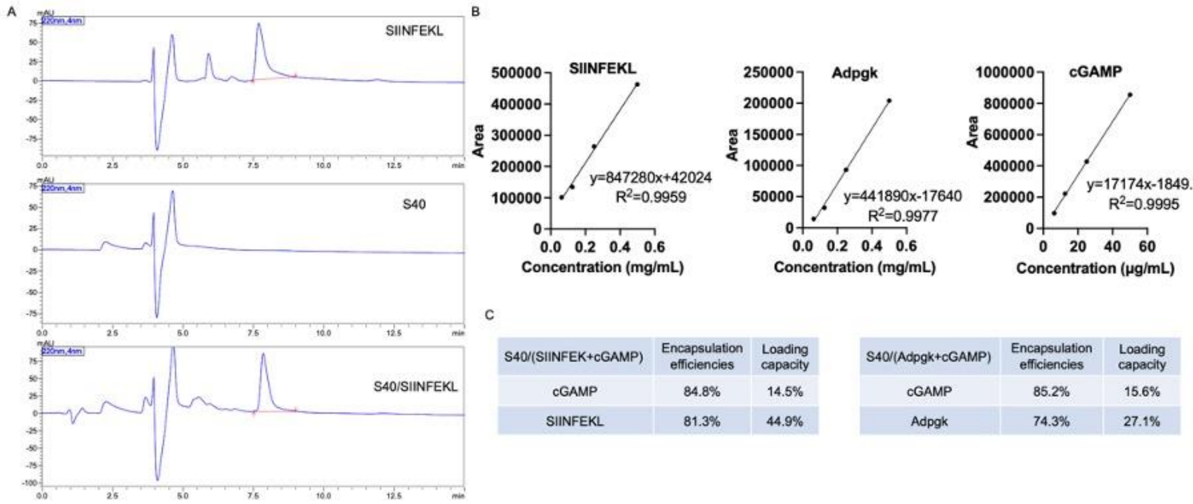

S40/(SIINFEK+cGAMP)	Encapsulation efficiencies	Loading capacity
cGAMP	84.8%	14.5%
SIINFEKL	81.3%	44.9%

S40/(Adpgk+cGAMP)	Encapsulation efficiencies	Loading capacity
cGAMP	85.2%	15.6%
Adpgk	74.3%	27.1%

Figure S11. (A) Representative HPLC chromatograms for the characterization of SIINFEKL loading in NPs. (B) The standard curves determined by HPLC to calculate the loading capacities of peptides and cGAMP. (C) Summary of encapsulation efficiencies and loading capacity of cGAMP and peptides in S40/(SIINFEKL+cGAMP) and S40/(Adpgk+cGAMP) NVs.

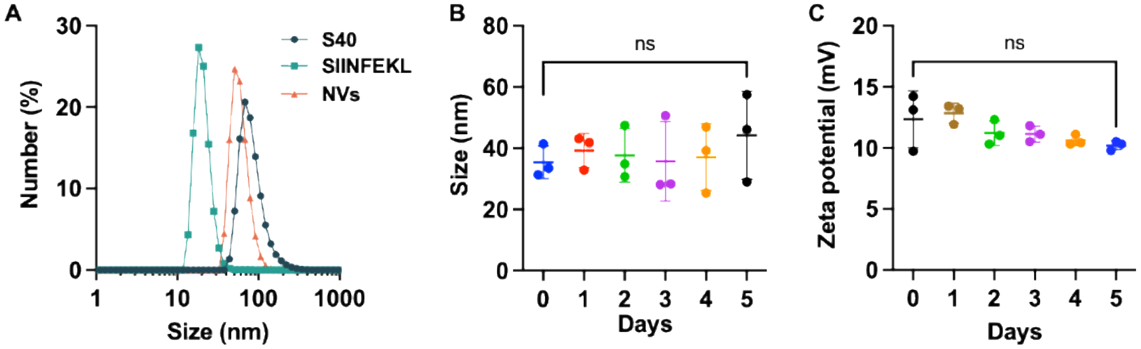

Figure S12. (A) The DLS graph of free SIINFEKL, S40 NPs, and NVs (S40 NPs loaded with SIINFEKL and cGAMP). (B, C) The stability of NVs in PBS was studied by monitoring the NV size distribution by DLS (B) and zeta potential (C) for 5 days.

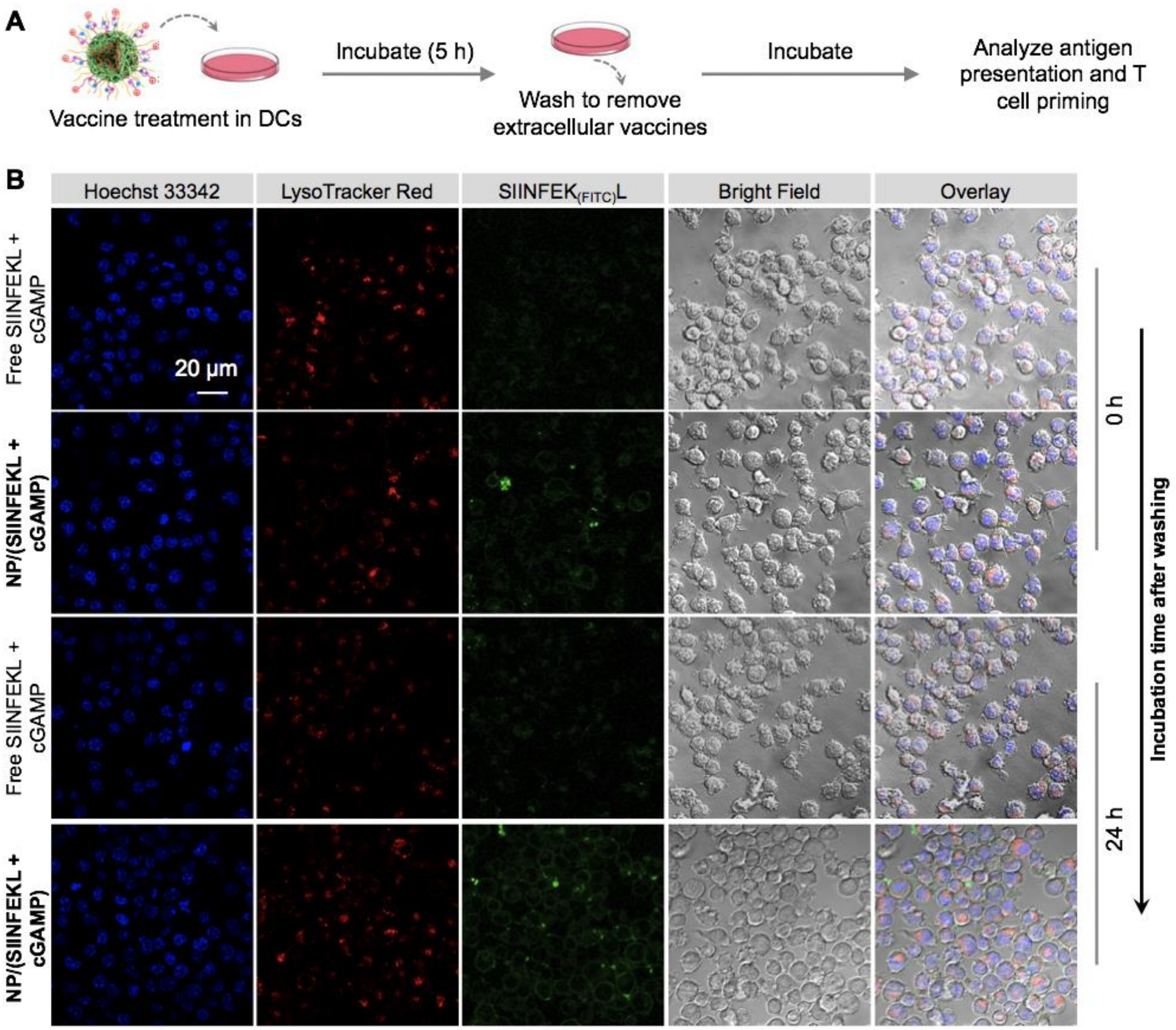

Figure S13. (A) Experiment design. (B) Confocal microscopy images of SIINFEK$_{(FITC)}$L presentation by DC2.4 cells after treatment with S40 loaded with cGAMP and SIINFEK$_{(FITC)}$L (NP/SIINFEKL+cGAMP) or free SIINFEK$_{(FITC)}$L with cGAMP (Free SIINFEKL+cGAMP). (cGAMP: 1 μg/mL; SIINFEK$_{FITC}$L: 2 μM).

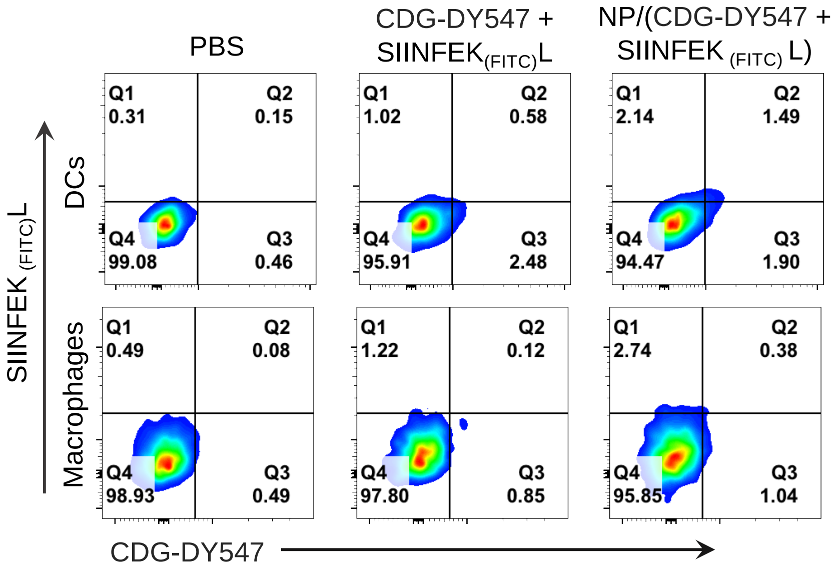

Figure S14. Flow cytometry plots showing the NVs codelivery of CDG and SIINFEKL into LN-residing DCs and macrophages, two primary intranodal APC subsets.

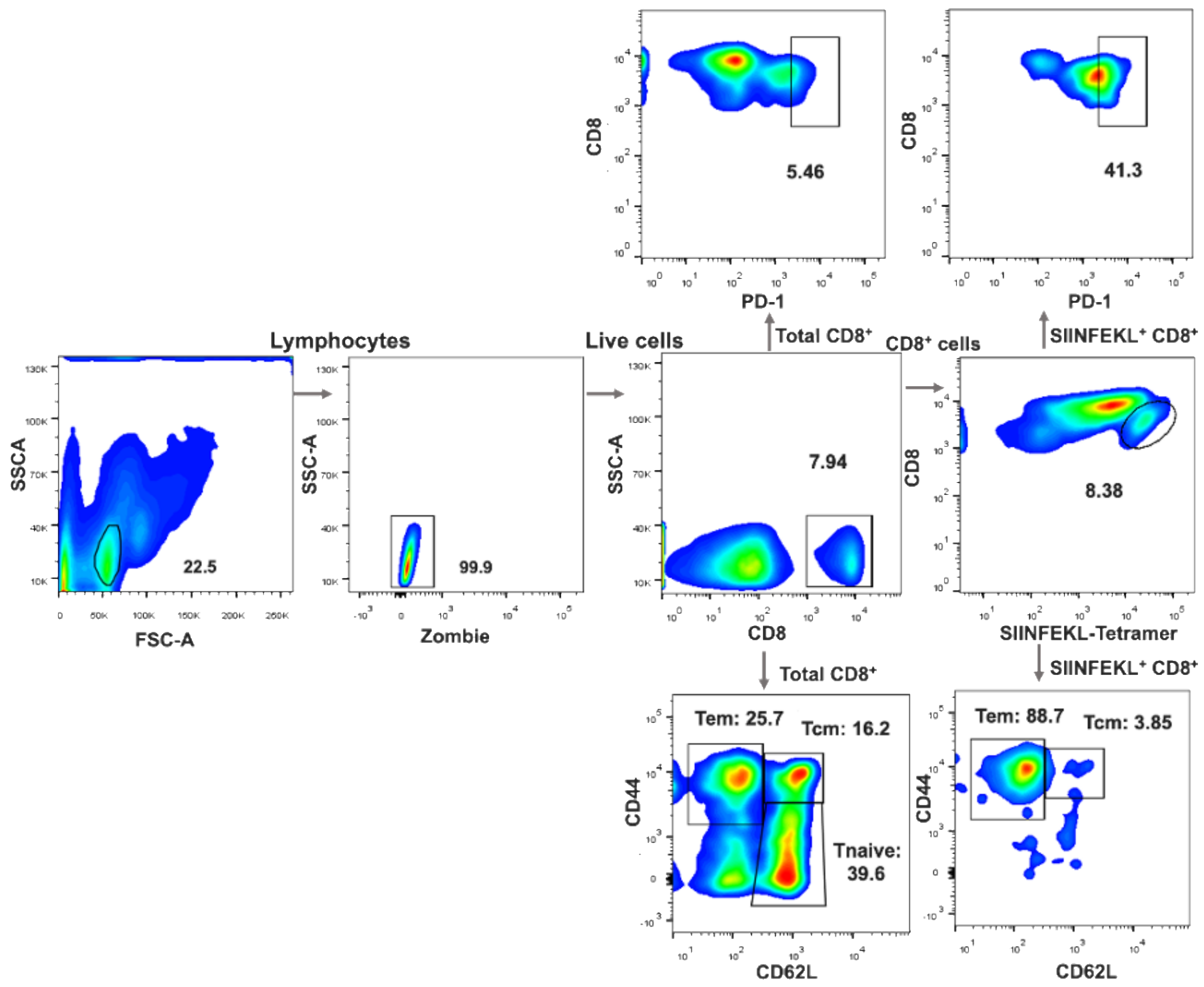

Figure S15. Gating tree for flow cytometric analysis of antigen-specific CD8^{+} T cell response via staining with a SIINFEKL/MHC-I tetramer and staining of effector memory T cells (T_{em}, CD62L^{-}CD44^{+}), central memory T cells (T_{cm}, CD62L^{high}CD44^{+}), and naive T cells ($T_{naïve}$, CD62L^{+}CD44^{-}).

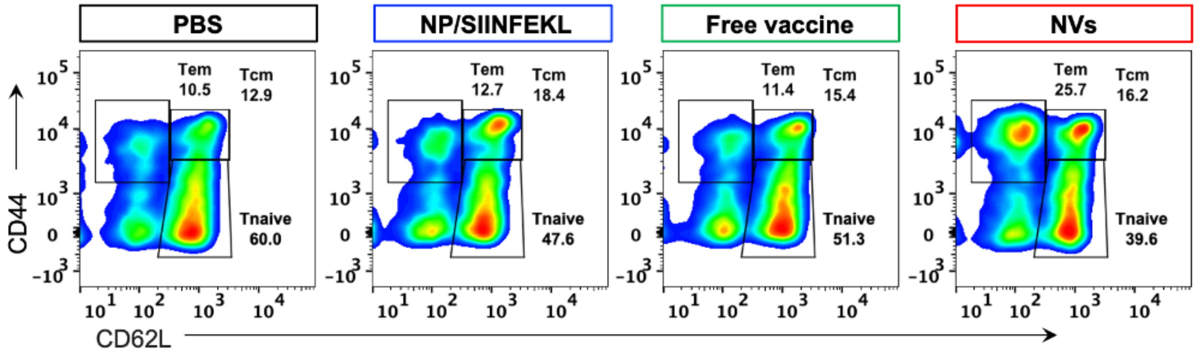

Figure S16. Representative flow cytometry plots of CD8^{+} memory T cells in mice immunized with NVs or controls (day 21).

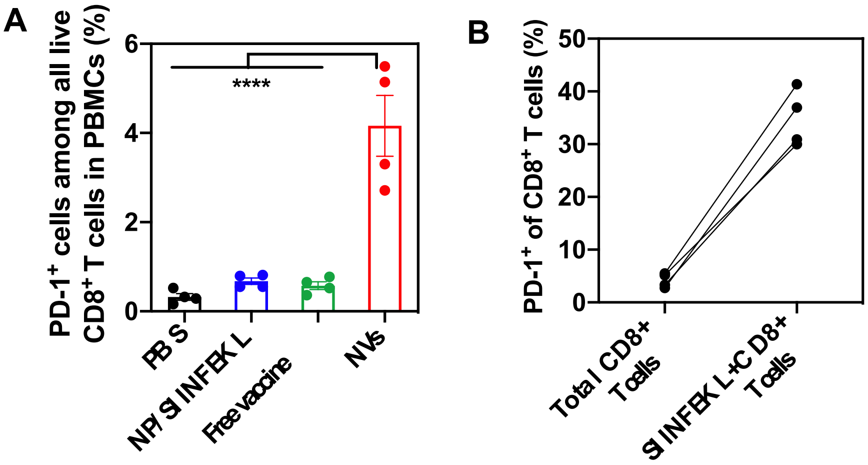

Figure S17. (A) The percentage of PD-1 positive cells among all PBMC CD8$^+$ T cells. (B) The frequency of PD-1 expression on SIINFEKL$^+$CD8$^+$ T cells and total CD8$^+$ T cells in peripheral blood on day 21.

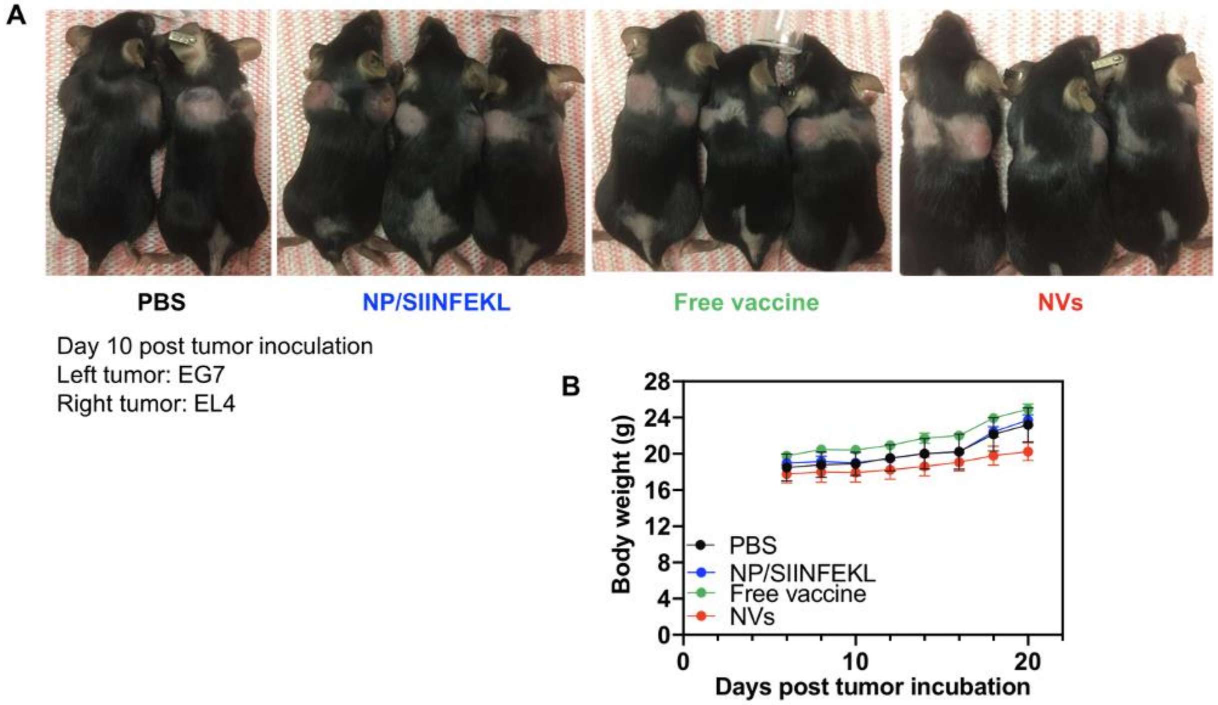

Figure S18. (A) Representative images of the tumors 10 days after inoculation of 8×10^5 EG7.OVA cells on the left shoulder and 8×10^5 EL4 cells on the right shoulder. (B) Mouse body weights over the course of treatment.

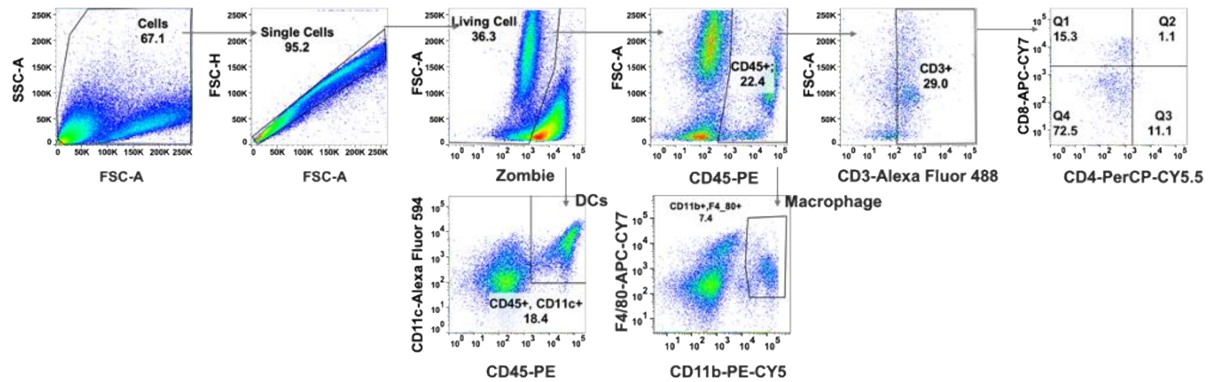

Figure S19. Gating tree for flow cytometric analysis of immune cell analysis in the tumor microenvironment.

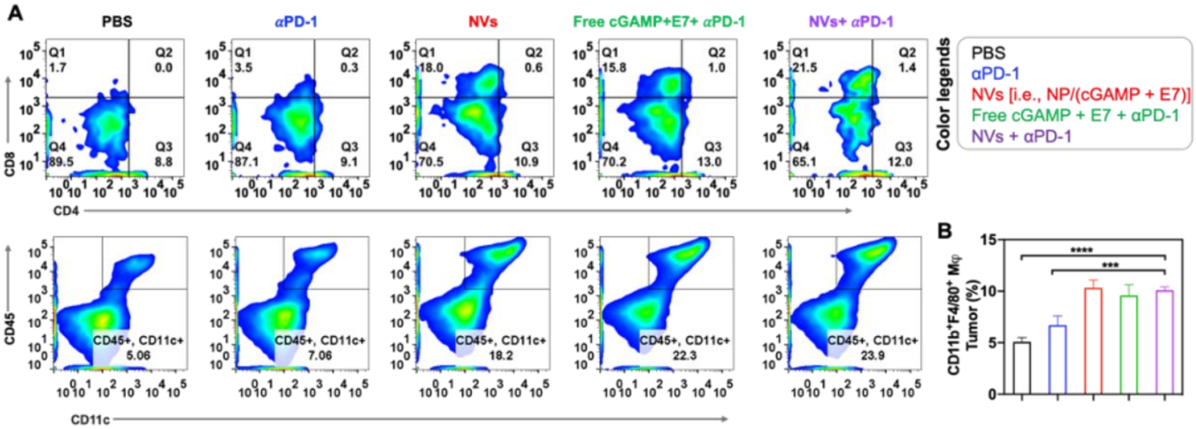

Figure S20. Flow cytometry results of Tumor macroenvironment. (A) Representative flow cytometry dot plot of tumor infiltrating $CD4^+$ and $CD8^+$ T cells and $CD45^+CD11c^+$ DC cells. (B) Quantification of the percentage of tumor infiltrating macrophages ($CD45^+CD11b^+F4/80^+$).

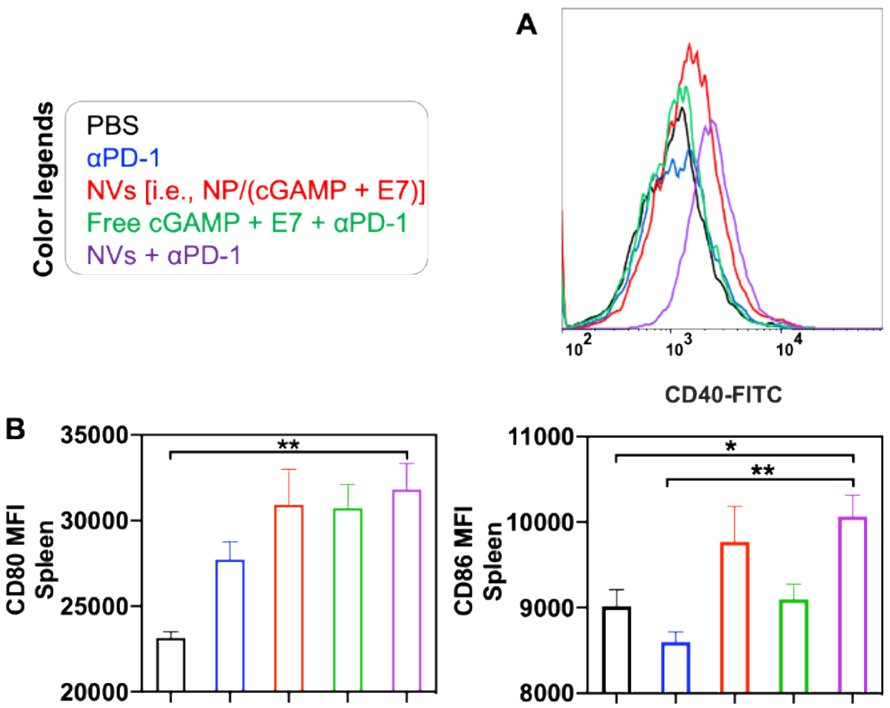

Figure S21. (A) Representative flow cytometry histograms of CD40 expression on DCs (gated on CD45$^+$CD11c$^+$ DCs). (B) Flow cytometric quantification of median fluorescence intensity (MFI) of CD80 and CD86 expression on DCs (gated on CD45$^+$CD11c$^+$ and CD45$^+$CD11c$^+$ DCs).

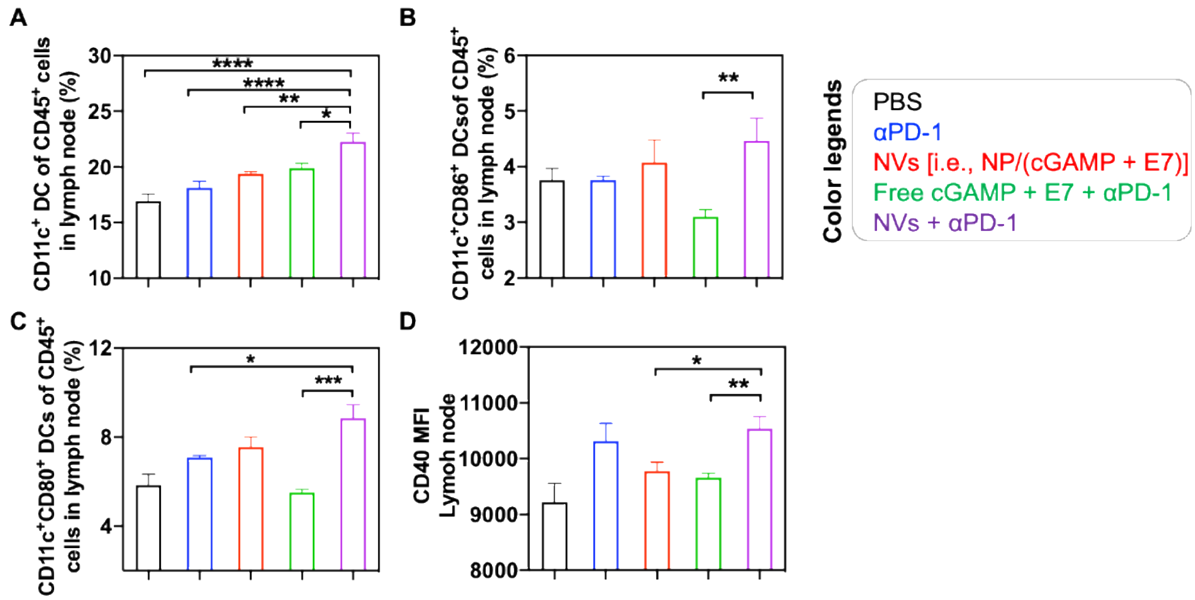

Figure S22. Percentage of CD45$^+$CD11c$^+$ DCs (A), CD45$^+$CD11c$^+$CD86$^+$ DCs (B) and CD45$^+$CD11c$^+$CD80$^+$ DCs (C) in the lymph nodes. (D) Quantification of median fluorescence intensity (MFI) of CD40 expression on DCs (CD45$^+$CD11c$^+$CD40$^+$) from lymph nodes.

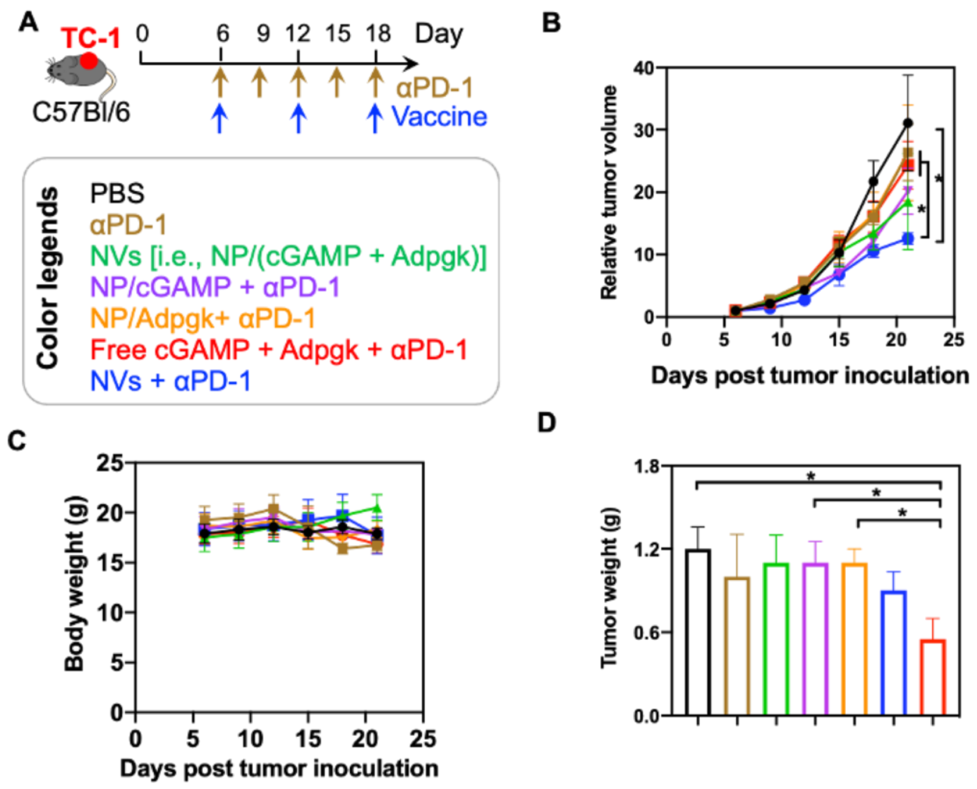

Figure S23. cGAMP/Adpgk-codelivering NVs mediated robust immunotherapy in combination with αPD-1 in MC38 tumor. (A) Study design. MC38 tumor progression (B) and mouse body weights (C) upon indicated treatment. The tumor weights (D) at the end of study on day 21.

Supplemental Tables

Table S1. Molecular weights of polymers calculated by ^{1}H NMR and GPC.

Polymers	DP$^{(1)}$	$M_n^{(2)}$ (Da)	GPC		
			M_n (Da)	M_w (Da)	PDI
mPEG-CHO	-	-	3248	3513	1.08
PEG(-alkynyl)-*b*-PDPA	17.8	5795	4211	6963	1.65
PDMA-N_3 (n=40)	35.4	5560	5158	6422	1.25
PEG(-*g*-PDMA)-*b*-PDPA (S40)	-	9119	6818	10791	1.58

(1) DP (degrees of polymerization): Determined from ^{1}H NMR spectrum.

(2) Determined from ^{1}H NMR spectrum.

Table S2. cGAMP loading capacity in S40 and S60 micelles.

	**S40**	**S40**	**S40**	**S40**	**S60**	**S60**	**S60**	**S60**
**Feeding**	5%	10%	15%	20%	5%	10%	15%	20%
**Loading Capacity**	1.25%	6.62%	8.58%	10.6%	2.3%	7.21%	10.15%	13.1%

Table S3. Primer sequences used in RT-PCR

**Primers**	**Sequences (5'-3')**
Tnf-α: Forward	5’-GGTGCCTATGTCTCAGCCTCTT-3’
Tnf-α: Reverse	5’-GCCATAGAACTGATGAGAGGGAG-3’
Cxcl10: Forward	5’-ATCATCCCTGCGAGCCTATCCT-3’
Cxcl10: Reverse	5’-GACCTTTTTTGGCTAAACGCTTTC-3’
Cxcl9: Forward	5’-CCTAGTGATAAGGAATGCACGATG-3’
Cxcl9: Reverse	5’-CTAGGCAGGTTTGATCTCCGTTC-3’
Il-6: Forward	5’-GAGGATACCACTCCCAACAGACC -3’
Il-6: Reverse	5’-AAGTGCATCATCGTTGTTCATACA - 3’
Ifn-β: Forward	5′-CGAGCAGAGATCTTCAGGAAC-3′
Ifn-β: Reverse	5′-TCACTACCAGTCCCAGAGTC-3′
Nos2: Forward	5’-TGCATGGACCAGTATAAGGCAAGC-3’
Nos2: Reverse	5’-GCTTCTGGTCGATGTCATGAGCAA-3’
Mrc1: Forward	5’-GTTCACCTGGAGTGATGGTTCTC-3’
Mrc1: Reverse	5’-AGGACATGCCAGGGTCACCTTT-3’
Ym1: Forward	5’-GGGCATACCTTTATCCTGAG-3’
Ym1: Reverse	5’-CCACTGAAGTCATCCATGTC-3’
Arg1: Forward	5’-CAGAAGAATGGAAGAGTCAG-3’
Arg1: Reverse	5’-CAGATATGCAGGGAGTCACC-3’
Gapdh: Forward	5′-CTTTGTCAAGCTCATTTCCTGG-3
Gapdh: Reverse	5′-TCTTGCTCAGTGTCCTTGC-3′
